# Trends and Racial‐Geographic Disparities in Coexisting Coronary Artery Disease (CAD) and Heart Failure (HF) Related Mortality Among U.S. Adults, 1999–2024

**DOI:** 10.1002/hsr2.72635

**Published:** 2026-06-09

**Authors:** Sahil Bhagia, Muhammad Salman Mustafa, Daniyal Khalid, Zainab Mazhar Baig, Noor Ahmed, Muhammad Mussawir Khuhro, Hasan Nazeer Khan, Duaa Mubashir, Sara Arsalan, Muhammad Bilal Khan, Hasibullah Aminpoor

**Affiliations:** ^1^ Department of Medicine and Surgery Dow University of Health Sciences Karachi Sindh Pakistan; ^2^ Department of Medicine and Surgery Jinnah Sindh Medical University Karachi Sindh Pakistan; ^3^ Resident Physician, Department of Internal Medicine Rabia Balkhi National Complex Hospital Kabul Afghanistan; ^4^ Faculty of Medicine Kabul University of Medical Sciences “Abu Ali Ibn Sina” Kabul Afghanistan

**Keywords:** coronary artery disease, health status disparities, heart failure, mortality trends, United States

## Abstract

**Background:**

Coronary artery disease (CAD) and heart failure (HF) frequently coexist, yet national mortality trends capturing both conditions together remain understudied. We examined U.S. mortality trends where CAD and HF coexisted from 1999 to 2024.

**Methods:**

We analyzed CDC WONDER multiple‐cause‐of‐death data for adults ≥ 25 years to identify deaths with coexisting CAD and HF. Age‐adjusted mortality rates (per 100,000) were standardized to the 2000 U.S. population. Joinpoint regression estimated annual percent changes, and mortality was stratified by demographics, region, and clinical presentation.

**Results:**

From 1999 to 2024, 2,930,567 deaths involved coexisting CAD and HF, with 32.82% occurring in inpatient settings. The AAMR declined significantly from 71.6 in 1999 to 43.7 in 2024. Men had higher mortality than women (67.5 vs. 39.8), although declines were steeper in women. White adults had the highest AAMR (52.6), while Asian or Pacific Islanders had the lowest (25.0). The Midwest recorded the highest mortality (54.6), and the Northeast the lowest (47.1). Mortality was higher in rural areas than in urban areas (63.6 vs. 49.5). Older adults had the greatest burden (240.9), whereas younger adults showed increasing trends after 2010. Chronic ischemic cardiomyopathy recorded higher mortality as compared to acute myocardial infarction (9.85 vs. 4.41), although the decline was greater for chronic infarctions. State‐level variation was notable, with West Virginia and Oklahoma consistently among the highest.

**Conclusion:**

Mortality involving coexisting CAD and HF declined overall but showed persistent demographic and geographic disparities. Targeted prevention strategies are needed to reduce the ischemic HF burden in high‐risk populations.

## 1 Introduction

Coronary artery disease (CAD) and heart failure (HF) remain major causes of cardiovascular morbidity and mortality in the United States. CAD, characterized by atherosclerotic narrowing of coronary arteries, reduces myocardial blood supply and is a leading cause of HF through myocardial ischemia, infarction, and progressive ventricular remodeling [1, 2]. HF affects over six million Americans aged ≥ 20 years, with prevalence projected to reach 8.7 million by 2030 [1, 2], and remains a leading cause of hospitalization among older adults.

Despite advances in acute coronary syndrome management guided by AHA/ACC/HFSA guidelines, CAD continues to drive long‐term cardiovascular mortality [3]. Mortality after acute myocardial infarction (AMI) has declined due to early revascularization, widespread statin use, and improved secondary prevention [3, 4, 5, 6]; however, better survival has increased the population living with chronic myocardial injury, contributing to ischemic cardiomyopathy and HF [7]. The pathophysiological link between CAD and HF involves myocardial fibrosis, neurohormonal activation, infarction‐induced ventricular remodeling, and progressive loss of contractility [8]. These processes result in structural and functional deterioration of the myocardium, a hallmark of ischemic HF, which carries substantial societal and healthcare burdens due to frequent hospitalizations, long‐term disability, and high mortality. Rising cardiometabolic risk factors, including obesity and type 2 diabetes components of the cardiovascular‐kidney‐metabolic syndrome, further elevate CAD and HF risk [9]. Although ischemic heart disease mortality has declined nationally, improvements are uneven across age, sex, race, ethnicity, and geography [10].

Despite the well‐established clinical relationship between CAD and HF, most national mortality analyses evaluate these conditions independently using the underlying cause of death, which may underestimate the true burden of ischemic HF. Ischemic HF arising from coronary atherosclerosis, myocardial injury, and progressive ventricular dysfunction is a major driver of hospitalizations, healthcare costs, and premature mortality. A multiple‐cause‐of‐death framework provides a more comprehensive assessment of deaths in which CAD and HF coexist. Accordingly, this study examines temporal trends and sociodemographic disparities in U.S. mortality from 1999 to 2024 using CDC WONDER multiple‐cause‐of‐death data and high‐sensitivity ICD‐10 coding, highlighting the need for targeted clinical and public health strategies to prevent avoidable cardiovascular deaths.

## 2 Methodology

### 2.1 Data Extraction

We conducted a retrospective analysis of U.S. mortality data obtained from the Centers for Disease Control and Prevention (CDC) Wide‐Ranging Online Data for Epidemiologic Research (WONDER) Multiple Cause‐of‐Death database [11]. This database compiles death certificate information reported by all U.S. states and territories and maintained by the National Center for Health Statistics. Mortality data were extracted for individuals aged 25 years or older between January 1, 1999 and December 31, 2024.

The CDC WONDER database suppresses cells with death counts fewer than 10 to protect confidentiality; in this study, all reported death counts met the minimum threshold, so no suppression occurred, and all strata were included in the analyses.

The primary outcome of interest was deaths involving both HF and CAD recorded on the death certificate. HF was identified using International Classification of Diseases, Tenth Revision (ICD‐10) code I50 and Hypertensive heart disease with (congestive) HF (I11.0), the latter included to maximize case ascertainment given its frequent coexistence with CAD. We acknowledge, however, that I11.0 may capture non‐ischemic HF cases; the implications of this are discussed in the Limitations. CAD was defined using ICD‐10 codes I20 through I25. These conditions were identified when listed as causes of death anywhere on the death certificate, indicating their coexistence as comorbid contributors to mortality. ICD‐10 definitions were applied according to international and U.S. coding standards [12].

Since CDC WONDER provides publicly available de‐identified data, this study was exempt from Institutional Review Board approval and was conducted in accordance with the Strengthening the Reporting of Observational Studies in Epidemiology guidelines [13].

### 2.2 Stratification

Mortality data were stratified by calendar year (1999–2024), sex (male, female), age group (25–44, 45–64, 65+), race/ethnicity (Hispanic or Latino, Asian or Pacific Islander, American Indian or Alaska Native, Black Adults or African American, and White), and geographic characteristics. The National Center for Health Statistics Urban–Rural Classification Scheme [14] was used to assess the population by metropolitan (large central, large fringe, medium, and small metropolitan) and non‐metropolitan areas and U.S. Census regions (Northeast, Midwest, South, and West) according to the U.S Census Bureau Definitions [15]. Race and ethnicity classifications correspond to those recorded on death certificates in accordance with Office of Management and Budget standards [16]. Place of death, categorized as a medical facility, home, hospice, or nursing home/long‐term care facility, was obtained for descriptive reporting. Urbanization analyses were restricted to 1999–2020 due to the application of the 2013 NCHS Urban–Rural Classification Scheme, which was only linked to CDC WONDER mortality data through 2020. Data for these stratifications beyond 2020 were therefore unavailable for this analysis.

### 2.3 Statistical Analysis

Crude mortality rates (CMRs) and age‐adjusted mortality rates (AAMRs) per 100,000 population were calculated for deaths involving coexisting CAD and HF between 1999 and 2024. CMRs were derived by dividing annual death counts by the corresponding U.S. population. AAMRs were computed by standardizing to the 2000 U.S. standard population, enabling comparisons across time and demographic groups while accounting for age structure differences [17]. Temporal trends in CMRs and AAMRs were evaluated using the Joinpoint Regression Program (Version 4.9.0.0, National Cancer Institute) [18]. Log‐linear segmented regression models identified significant changes in trends over time. Autocorrelation was assessed within the program, and permutation tests with default settings determined the optimal number of joinpoints [19]. Annual percent changes were calculated for each segment using the parametric method and are presented with 95% confidence intervals (CIs). Statistical significance was assessed using two‐tailed *t*‐tests, with *p* values less than 0.05 considered significant.

### 2.4 Subgroup Analysis

A subgroup analysis was conducted to distinguish deaths occurring during acute ischemic events from those associated with chronic ischemic myocardial injury. Two categories were defined based on ICD‐10 codes recorded on death certificates. The AMI group included deaths involving ICD‐10 code I21 AMI. The chronic ischemic cardiomyopathy group included deaths involving ICD‐10 codes I25.2 (Old Myocardial Infarction) and I25.5 (Ischemic Cardiomyopathy). These codes were selected to represent chronic sequelae of ischemic heart disease, including prior infarction and long‐term myocardial dysfunction. For each subgroup, AAMRs and temporal trends were analyzed using Joinpoint regression, with APCs calculated as above for other groups.

### 2.5 Sensitivity Analysis

Two sensitivity analyses were conducted. First, to account for potential pandemic‐era reporting effects, deaths with COVID‐19 (U07.1) were excluded, and AAMRs were recalculated for 2018–2024. Second, to assess the influence of including I11.0, HF was restricted to I50 only, and trends were recalculated through 1999–2024. Results were compared with the primary analysis to evaluate robustness.

## 3 Results

**Figure 1:**
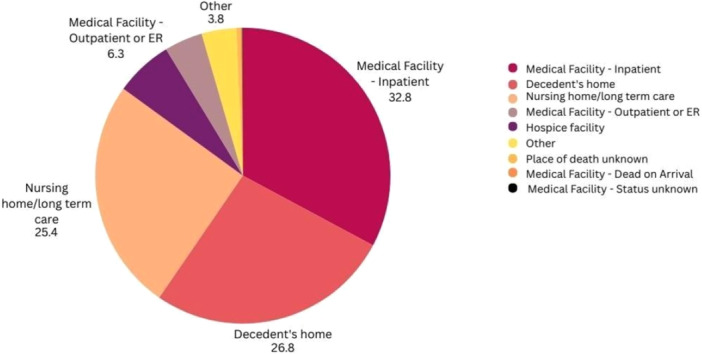
Coronary artery disease (CAD) and heart failure (HF) related mortality in adult patients in the US, 1999–2024, by place of death.

From 1999 to 2024, a total of 2,930,567 deaths occurred in the United States where CAD and HF coexisted as comorbid contributors. Among them, 47.45% were females, accounting for 1,390,415 deaths, and 52.55% were males, accounting for 1,540,152 deaths (Table S1). Information on the place of death revealed that a substantial proportion of deaths occurred in inpatient medical facilities, with additional deaths occurring in outpatient or emergency departments, and a smaller fraction arriving dead on arrival or in medical facilities with unknown status. A notable number of deaths occurred at the decedent's home, in hospice facilities, and in nursing homes or long‐term care facilities. Other locations accounted for a smaller share of deaths, and in some cases, the place of death was unknown (Table S2, Figure 1).

Mortality varied substantially by sex, race, census region, urbanization status, age group, state, and clinical presentation (acute vs. chronic ischemic heart disease). Temporal patterns in mortality and differences across population subgroups are described in the following sections.

### 3.1 Annual Mortality Trends

**Figure 2:**
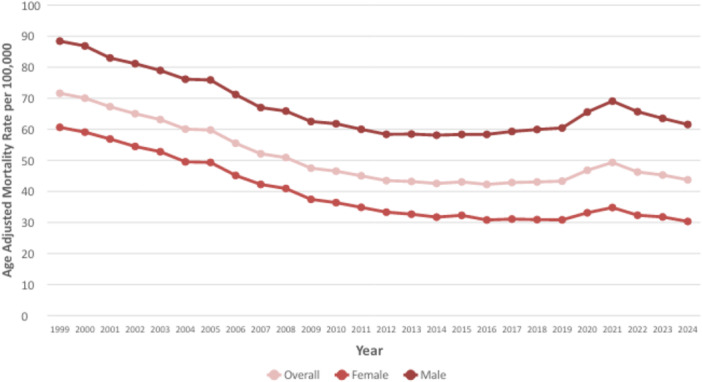
Coronary artery disease (CAD) and heart failure (HF) related mortality in adult patients in the US, 1999–2024, by overall and sex.

AAMRs per 100,000 population demonstrated a significant overall decline across the study period. The AAMR decreased from 71.6 in 1999 to 43.7 in 2024, representing a substantial overall reduction (AAPC −2.05%; 95% CI: −2.67 to −1.43; *p* < 0.001) (Table S4). Trend analysis showed a significant decline from 1999 to 2012 (APC −3.97%; 95% CI: −4.21 to −3.73), a nonsignificant plateau from 2012 to 2018 (APC −0.31%; 95% CI: −1.42 to 0.81), a significant increase from 2018 to 2021 (APC 4.72%; 95% CI: 0.01–9.65), followed by a significant decline from 2021 to 2024 (APC −3.66%; 95% CI: −5.85 to −1.43) (Tables S1 and S3, Figure 2).

## 4 Demographic Differences

### 4.1 Sex Trends and Disparities

Men had higher AAMRs than women throughout the study duration (overall AAMR men: 67.5; 95% CI: 67.0–68.1; women: 39.8; 95% CI: 39.5–40.2).

Among females mortality rates showed a significant decline in the three stages; from 1999 to 2005 (APC −3.85%; 95% CI: −4.44 to −3.25), from 2005 to 2011 (APC −5.70%; 95% CI: −6.57 to −4.82), and from 2011 to 2018 (APC −1.63%; 95% CI: −2.37 to −0.88), followed by a nonsignificant increase from 2018 to 2021 (APC 4.25%; 95% CI: −0.08 to 8.76) and a significant decline from 2021 to 2024 (APC −4.11%; 95% CI: −6.10 to −2.09).

Males demonstrated a significant decline from 1999 to 2012 (APC −3.30%; 95% CI: −3.51 to −3.09), a nonsignificant plateau from 2012 to 2018 (APC 0.34%; 95% CI: −0.56 to 1.24), a significant increase from 2018 to 2021 (APC 5.05%; 95% CI: 1.32–8.92), and a significant decline from 2021 to 2024 (APC −3.60%; 95% CI: −5.27 to −1.90).

The overall AAPC demonstrated a significant long‐term decline in CAD/HF‐related mortality for both sexes, with a steeper decrease observed among women (AAPC: −2.77%; 95% CI: −3.35 to −2.19) compared with men (AAPC: −1.50%; 95% CI: −1.99 to −1.01) (Tables S3 and S4 Figure 2).

### 4.2 Age Group Trends and Disparities

When stratifying adults by age group, Older Adults consistently displayed the highest mortality, followed by Middle Age Adults, and Young Adults (overall AAMR Older Adults: 240.9; 95% CI: 239.4–242.4; Middle Age Adults: 11.0; 95% CI: 10.8–11.2; Young Adults: 0.6; 95% CI: 0.6–0.7).

Among Older Adults, the mortality rate decreased from 1999 to 2012 (APC: −4.03; 95% CI: −4.28 to −3.78), followed by a period of nonsignificant plateau from 2012 to 2018 (APC: −0.68; 95% CI: −1.83 to 0.48), a nonsignificant increase from 2018 to 2021 (APC: 4.33; 95% CI: −0.63 to 9.54), and a subsequent decline from 2021 to 2024 (APC: −3.70; 95% CI: −5.96 to −1.39).

Among Middle Age Adults, mortality declined from 1999 to 2011 (APC: −3.52; 95% CI: −3.77 to −3.27), followed by a significant increase from 2011 to 2018 (APC: 2.96; 95% CI: 2.22–3.71), a further steep significant increase from 2018 to 2021 (APC: 8.38; 95% CI: 4.40–12.51), and a subsequent nonsignificant decline from 2021 to 2024 (APC: −3.22; 95% CI: −5.01 to −1.41).

Among Young Adults, mortality showed nonsignificant fluctuations from 1999 to 2010, followed by a significant increase from 2010 to 2022 (APC: 5.81; 95% CI: 4.12–7.53), and a nonsignificant decline from 2022 to 2024 (APC: −6.23; 95% CI: −23.23 to 14.54).

**Figure 3:**
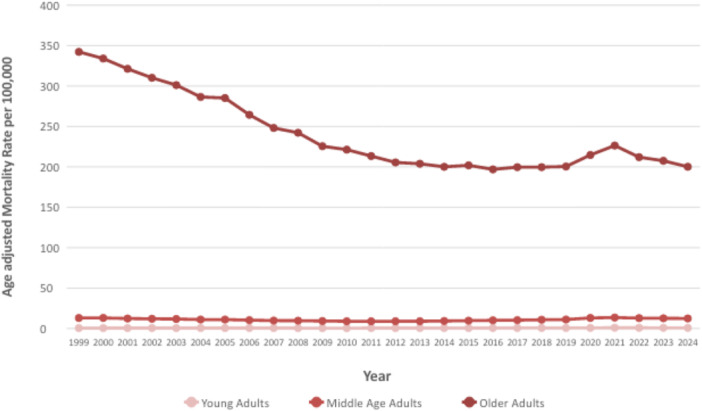
Coronary artery disease (CAD) and heart failure (HF) related mortality in adult patients in the US, 1999–2024, by age group.

Over the 1999‐2024 period, long‐term trends in CAD/HF‐related mortality differed markedly by age group. Mortality rates among Young Adults (AAPC: 1.59%; 95% CI: −1.08 to 4.34) and Middle‐Aged Adults (AAPC: −0.33%; 95% CI: −0.84 to 0.18) showed no statistically significant change. In contrast, a significant decline was observed among Older Adults (AAPC: −2.22%; 95% CI: −2.87 to −1.57) (Tables S3 and S5 Figure 3).

### 4.3 Racial Trends and Disparities

When stratified by race/ethnicity, White adults had the highest AAMR, followed by Black or African American adults, American Indian or Alaska Native adults, Hispanic or Latino adults, and Asian or Pacific Islander adults (overall AAMR White: 52.6; 95% CI: 52.3–52.9; Black: 45.5; 95% CI: 44.5–46.5; American Indian or Alaska Native: 40.0; 95% CI: 36.4–43.6; Hispanic or Latino: 37.5; 95% CI: 36.4–38.5; Asian or Pacific Islander: 25.0; 95% CI: 23.8–26.1).

Among White individuals, mortality rates showed a significant decline from 1999 to 2012 (APC −3.93%; 95% CI: −4.18 to −3.69), a nonsignificant plateau from 2012 to 2018 (APC −0.25%; 95% CI: −1.35 to 0.87), a significant increase from 2018 to 2021 (APC 4.90%; 95% CI: 0.04 to 9.99), followed by a significant decline from 2021 to 2024 (APC −3.34%; 95% CI: −5.58 to −1.06).

black adults showed a significant decline from 1999 to 2012 (APC −3.73%; 95% CI: −4.08 to −3.37), a nonsignificant plateau from 2012 to 2018 (APC 0.34%; 95% CI: −1.18 to 1.89), a significant increase from 2018 to 2021 (APC 6.20%; 95% CI: 0.07–12.70), followed by a significant decline from 2021 to 2024 (APC −3.95%; 95% CI: −6.70 to −1.11).

American Indian or Alaska Native adults demonstrated a steady significant decline across the entire period from 1999 to 2024 (APC: −1.58; 95% CI: −2.00 to −1.15).

Hispanics or Latino demonstrated a significant decline from 1999 to 2012 (APC −4.25%; 95% CI: −4.57 to −3.93), a nonsignificant plateau from 2012 to 2018 (APC −0.37%; 95% CI: −1.58 to 0.84), a significant increase from 2018 to 2021 (APC 5.06%; 95% CI: 0.21–10.15), followed by a significant decline from 2021 to 2024 (APC − 7.01%; 95% CI: −9.12 to −4.85).

Asian or Pacific Islanders showed a significant decline from 1999 to 2014 (APC −3.82%; 95% CI: −4.31 to −3.33), a nonsignificant increase from 2014 to 2021 (APC 1.30%; 95% CI: −0.25 to 2.88), followed by a significant decline from 2021 to 2024 (APC −4.93%; 95% CI: −8.82 to −0.87).

**Figure 4:**
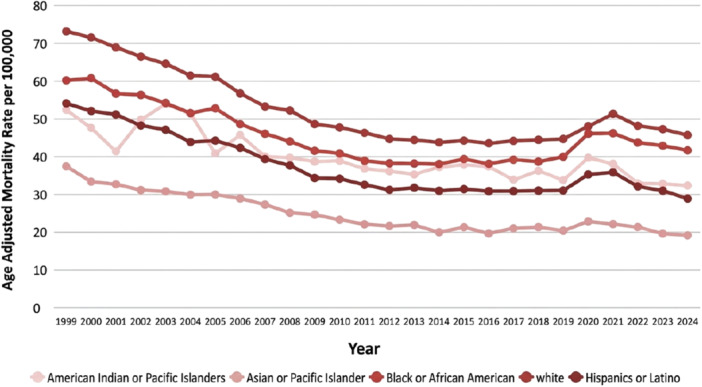
Coronary artery disease (CAD) and heart failure (HF) related mortality in adult patients in the US, 1999–2024, by race.

From 1999 to 2024, CAD/HF‐related mortality declined significantly among all racial/ethnic groups, with the steepest declines observed among Hispanic or Latino adults (AAPC: −2.59%; 95% CI: −3.23 to −1.95) and Asian or Pacific Islander adults (AAPC: −2.55%; 95% CI: −3.21 to −1.88), followed by White adults (AAPC: −1.96%; 95% CI: −2.59 to −1.33), Black adults (AAPC: −1.64%; 95% CI: −2.45 to −0.83), and American Indian or Alaska Native adults (AAPC: −1.58%; 95% CI: −2.00 to −1.15) (Tables S3 and S6 Figure 4).

## 5 Regional Differences

### 5.1 Census Region Trends and Disparities

Adults living in the Midwest had the highest mortality rates, followed by the South, the West, and the Northeast (overall AAMR Midwest: 54.6; 95% CI: 53.9–55.2; South: 51.3; 95% CI: 50.8–51.8; West: 50.3; 95% CI: 49.7–51.0; Northeast: 47.1; 95% CI: 46.5–47.7).

In the Midwest, a significant decline was observed from 1999 to 2012 (APC −3.93%; 95% CI: −4.18 to −3.67), followed by a nonsignificant plateau from 2012 to 2018 (APC 0.03%; 95% CI: −1.17 to 1.25), a significant increase from 2018 to 2021 (APC 5.33%; 95% CI: 0.14–10.80), and a significant decline from 2021 to 2024 (APC −2.78%; 95% CI: −5.16 to −0.35).

In the South, significant declines were observed from 1999 to 2005 (APC −3.22%; 95% CI: −3.73 to −2.71) and from 2005 to 2011 (APC −4.94%; 95% CI: −5.64 to −4.24), followed by a nonsignificant plateau from 2011 to 2018 (APC 0.05%; 95% CI: −0.50 to 0.60), a significant increase from 2018 to 2021 (APC 6.00%; 95% CI: 2.89 to 9.20), and a significant decline from 2021 to 2024 (APC −3.14%; 95% CI: −4.49 to −1.76).

In the West, significant declines were observed from 1999 to 2003 (APC −2.74%; 95% CI: −3.90 to −1.56) and from 2003 to 2012 (APC −4.16%; 95% CI: −4.58 to −3.75), followed by a nonsignificant plateau from 2012 to 2018 (APC −0.53%; 95% CI: −1.40 to 0.35), a significant increase from 2018 to 2021 (APC 4.22%; 95% CI: 0.47–8.11), and a significant decline from 2021 to 2024 (APC −4.64%; 95% CI: −6.34 to −2.92).

In the Northeast, significant declines were observed in three stages; from 1999 to 2005 (APC −3.49%; 95% CI: −4.12 to −2.86), from 2005 to 2009 (APC −5.77%; 95% CI: −7.73 to −3.77), and from 2009 to 2017 (APC −2.10%; 95% CI: −2.69 to −1.51), followed by a nonsignificant increase from 2017 to 2021 (APC 1.40%; 95% CI: −0.79 to 3.63) and a significant decline from 2021 to 2024 (APC −4.86%; 95% CI: −6.99 to −2.68).

**Figure 5:**
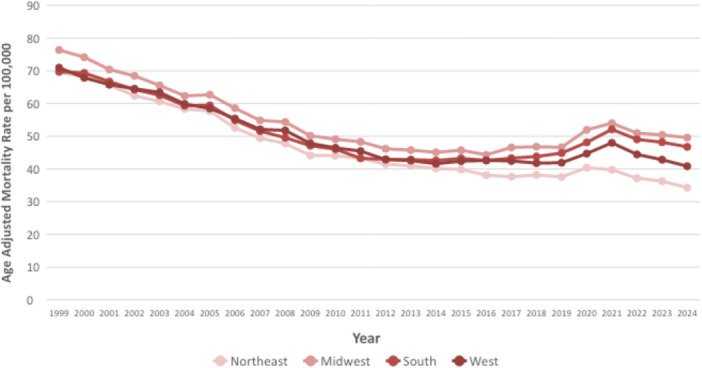
Coronary artery disease (CAD) and heart failure (HF) related mortality in adult patients in the US, 1999–2024, by census region.

Over the entire study period (1999‐2024), all four census regions experienced significant overall declines in CAD/HF‐related mortality: Northeast (AAPC: −2.82%; 95% CI: −3.35 to −2.29), Midwest (AAPC: −1.78%; 95% CI: −2.45 to −1.10), South (AAPC: −1.66%; 95% CI: −2.08 to −1.24), and West (AAPC: −2.15%; 95% CI: −2.67 to −1.63) (Tables S3 and S7 Figure 5).

### 5.2 Urbanization Trends and Disparities

A significant variation in AAMR was observed when comparing Urban and Rural areas. Rural areas consistently had higher AAMR than urban areas throughout the study period (overall AAMR Rural: 63.6; 95% CI: 62.8–64.4; Urban: 49.5; 95% CI: 49.2–49.9).

The mortality rate for Rural areas decreased significantly from 1999 to 2013 (APC: −3.29; 95% CI: −3.55 to −3.04), followed by a nonsignificant plateau from 2013 to 2018 (APC: 0.71; 95% CI: −1.14 to 2.59), and a nonsignificant increase from 2018 to 2020 (APC: 4.97; 95% CI: −0.61 to 10.86). AAMR of Urban areas declined from 1999 to 2005 (APC: −3.44; 95% CI: −3.94 to −2.94), followed by a steeper significant decline from 2005 to 2009 (APC: −5.30; 95% CI: −6.82 to −3.76), a nonsignificant decline from 2009 to 2012 (APC: −3.00; 95% CI: −6.19 to 0.28), a nonsignificant plateau from 2012 to 2018 (APC: −0.49; 95% CI: −1.22 to 0.25), and an increase from 2018 to 2020 (APC: 4.39; 95% CI: 1.21–7.67).

**Figure 6:**
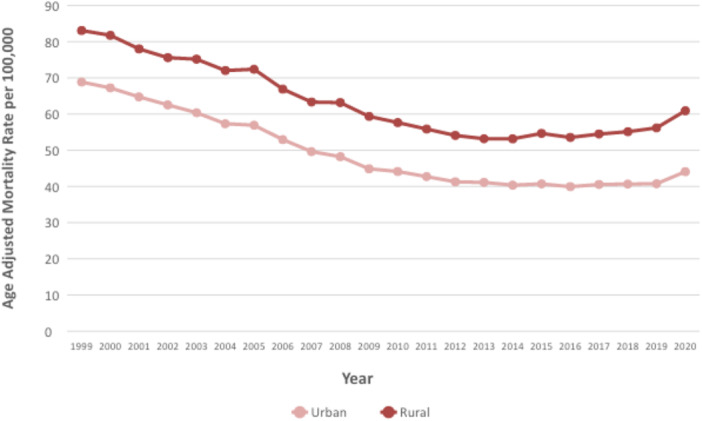
Coronary artery disease (CAD) and heart failure (HF) related mortality in adult patients in the US, 1999–2020, by urbanization.

Over the entire study period (1999–20), both urban and rural areas experienced significant overall declines in CAD/HF‐related mortality, with urban areas showing a steeper decline (AAPC: −2.18%; 95% CI: −2.75 to −1.61) compared to rural areas (AAPC: −1.59%; 95% CI: −2.22 to −0.96) (Tables S3 and S8 Figure 6).

### 5.3 State Trends and Disparities

AAMR differed substantially across U.S. states and the District of Columbia during the study period.

1999–2020: AAMRs ranged from a low of 30.38 (95% CI: 29.68–31.09) in Hawaii to a high of 76.92 (95% CI: 75.98–77.87) in West Virginia. States in the top 90th percentile included West Virginia (76.92; 95% CI: 75.98–77.87), Oklahoma (76.84; 95% CI: 76.12–77.56), Rhode Island (71.59; 95% CI: 70.41–72.77), and Tennessee (69.55; 95% CI: 69.01–70.08). In contrast, states in the bottom 10th percentile included Hawaii (30.38; 95% CI: 29.68–31.09), Alaska (36.13; 95% CI: 34.49–37.78), the District of Columbia (36.98; 95% CI: 35.68–38.27), and Virginia (38.67; 95% CI: 38.31–39.04).

**Figure 7:**
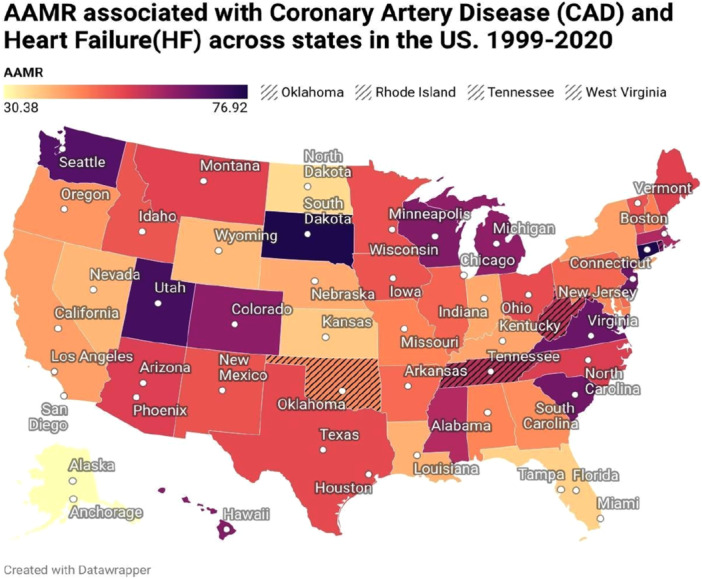
Coronary artery disease (CAD) and heart failure (HF) related mortality in adult patients in the US, 1999–2020, by states.

**Figure 8:**
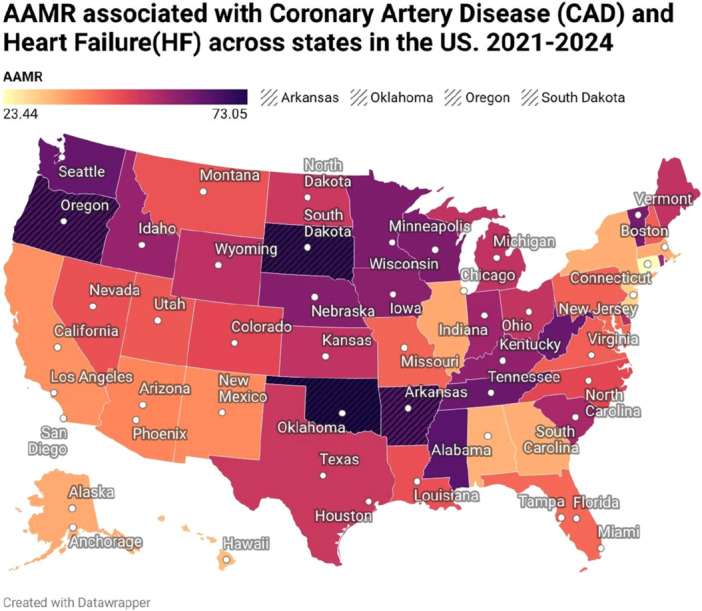
Coronary artery disease (CAD) and heart failure (HF) related mortality in adult patients in the US, 2021–2024, by states.

2021–2024: AAMRs ranged from a low of 23.44 (95% CI: 22.62–24.30) in Connecticut to a high of 73.05 (95% CI: 71.54–74.60) in Oklahoma. States in the top 90th percentile included Oklahoma (73.05; 95% CI: 71.54–74.60), Arkansas (66.14; 95% CI: 64.54–67.77), Mississippi (65.12; 95% CI: 63.47–66.80), and West Virginia (63.24; 95% CI: 61.34–65.19). States in the bottom 10th percentile included Connecticut (23.44; 95% CI: 22.62–24.30), New Jersey (29.60; 95% CI: 29.00–30.21), New York (34.24; 95% CI: 33.81–34.68), and Massachusetts (34.68; 95% CI: 33.94–35.44) (Table S9, Figures 7, 8).

### 5.4 Subgroup Analysis on the Basis of Clinical Presentation

#### 5.4.1 AMI vs. Chronic Ischemic Cardiomyopathy

When comparing AMI with chronic ischemic cardiomyopathy, distinct temporal patterns emerged. Chronic ischemic cardiomyopathy had an overall AAMR of 4.4 (95% CI: 4.3–4.5), while acute MI had an overall AAMR of 9.9 (95% CI: 9.7–10.0).

For chronic ischemic cardiomyopathy, mortality declined significantly from 1999 to 2013 (APC: −2.39; 95% CI: −2.76 to −2.01), followed by a slight significant increase from 2013 to 2021 (APC: 1.67; 95% CI: 0.61–2.74), and a nonsignificant decline from 2021 to 2024 (APC: −3.74; 95% CI: −7.47 to 0.14). For acute MI, mortality showed a significant steep decline from 1999 to 2011, with varying rates; from 1999 to 2002 (APC: −3.54; 95% CI: −5.27 to −1.78), from 2002 to 2011 (APC: −5.86; 95% CI: −6.28 to −5.45), followed by a slower nonsignificant decline from 2011 to 2018 (APC: −0.82; 95% CI: −1.57 to −0.06), an significant increase from 2018 to 2021 (APC: 5.70; 95% CI: 1.51–10.07), and a subsequent significant decline from 2021 to 2024 (APC: −4.88; 95% CI: −6.76 to −2.96).

**Figure 9:**
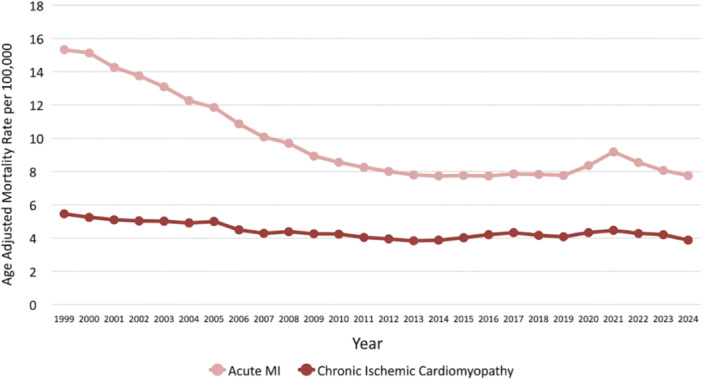
Coronary artery disease (CAD) and heart failure (HF) related mortality in adult patients in the US, 1999–2024, by clinical presentation.

Over the entire study period (1999–2024), both conditions showed significant overall declines, with acute MI demonstrating a steeper decline (AAPC: −2.74%; 95% CI: −3.29 to −2.17) compared to chronic ischemic cardiomyopathy (AAPC: −1.27%; 95% CI: −1.84 to −0.70) (Tables S3 and S10, Figure 9).

### 5.5 COVID‐19 Sensitivity Analysis

**Figure 10:**
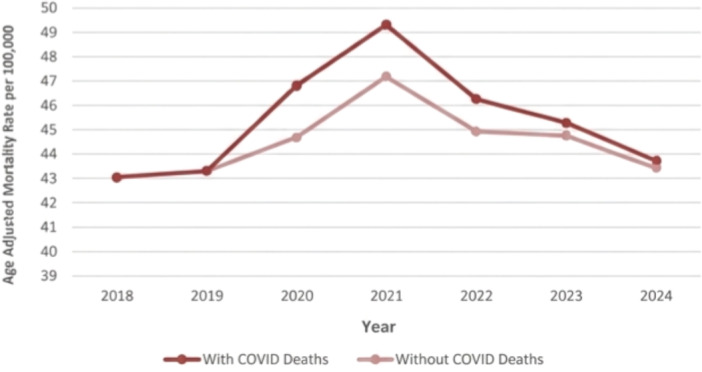
Coronary artery disease (CAD) and heart failure (HF) related mortality in adult patients in the US, 2018–2024, by sensitivity analysis; without COVID deaths vs. with COVID deaths.

To assess whether pandemic‐era death certification practices artificially inflated the 2018–2021 mortality spike, a sensitivity analysis was conducted excluding all deaths in which COVID‐19 (ICD‐10: U07.1) appeared anywhere on the death certificate. After exclusion of 5747 COVID‐19‐associated deaths in 2020 and 5576 in 2021, the AAMR in 2020 decreased from 46.81 to 44.68, and the 2021 AAMR decreased from 49.33 to 47.19, an attenuation of approximately 2.1 units (4.3%) at the peak. Importantly, the upward trend from 2018 to 2021 persisted in the sensitivity analysis (AAMR increase: +4.13 vs. +6.27 in the primary analysis), confirming that the observed spike reflects genuine cardiovascular deterioration rather than a coding artifact. By 2024, the sensitivity AAMR (43.43) closely approximated the primary analysis AAMR (43.74), indicating minimal COVID‐19 coding influence in later years (Table S11, Figure 10).

### 5.6 I11.0 Exclusion Sensitivity Analysis

**Figure 11:**
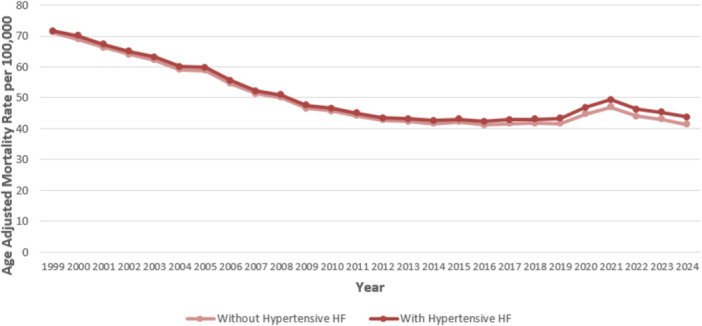
Coronary artery disease (CAD) and heart failure (HF) related mortality in adult patients in the US, 1999–2024, by sensitivity analysis; without hypertensive HF vs. with hypertensive HF.

To assess whether inclusion of ICD‐10 code I11.0 (hypertensive heart disease with HF) materially affected the primary findings, a sensitivity analysis was conducted restricting the HF definition to I50 codes only. Excluding I11.0 removed 57,928 deaths (2.0% of the primary cohort of 2,930,567). The overall AAMR in the sensitivity analysis was 50.23 compared with 51.14 in the primary analysis, a difference of less than 1 unit (1.8%). The direction and magnitude of all temporal trends were preserved: AAMR declined from 71.61 in 1999 to 41.57 in 2024 (vs. 71.62–43.74 in the primary analysis), the 2012–2018 plateau was maintained, and the 2018–2021 spike persisted (sensitivity AAMR peak: 47.27 vs. 49.33 in the primary analysis). These findings confirm that the inclusion of I11.0 did not meaningfully alter the study's conclusions, and that results are robust to this coding decision (Table S12, Figure 11).

## 6 Discussions

**Figure 12:**
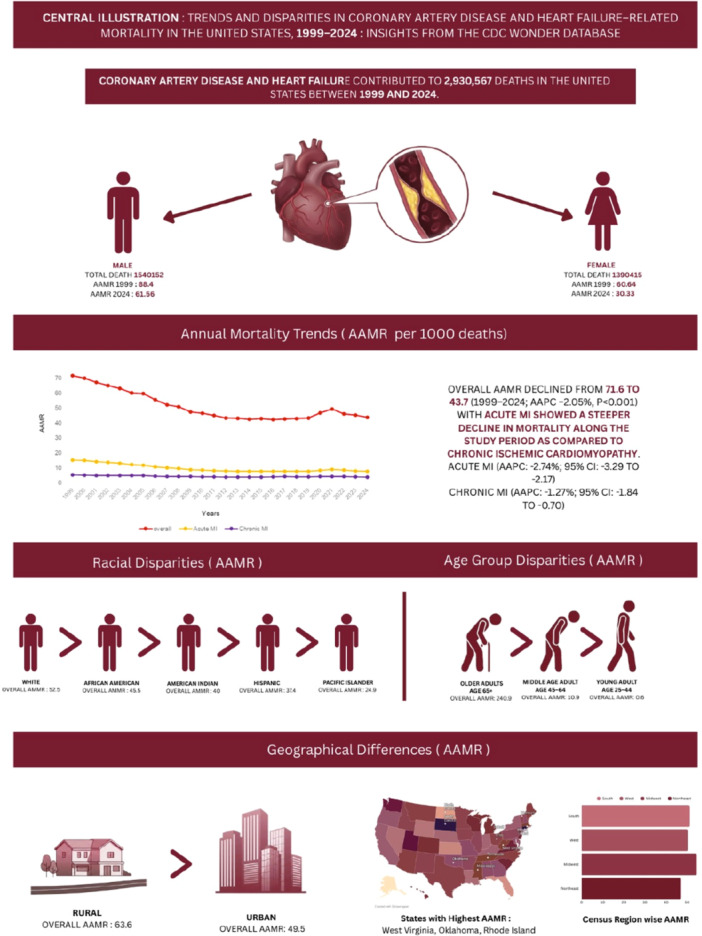
Central illustration.

In this nationwide analysis of mortality data derived from the CDC Multiple Cause of Death database, we examined long‐term temporal trends and sociodemographic disparities in mortality involving the coexistence of CAD and HF between 1999 and 2024. Over the 26‐year study period, more than 2.9 million deaths were identified in which CAD and HF were jointly listed as contributing causes of death, underscoring the substantial population‐level burden of ischemic HF in the United States. Several key findings emerged from this analysis. First, CAD‐HF mortality demonstrated a distinct temporal trajectory characterized by early declines during the early 2000s, followed by a period of stabilization and transient increases before more recent improvements. Second, marked disparities were observed across sex, race and ethnicity, age groups, geographic regions, and levels of urbanization, highlighting persistent inequities in cardiovascular outcomes. Third, considerable state‐level heterogeneity was observed, suggesting regional clustering of disease burden. Fourthly, divergent trends between acute ischemic events and chronic ischemic cardiomyopathy highlight a shifting clinical landscape in which improved survival after acute coronary syndromes is increasingly accompanied by long‐term HF‐related mortality (Figure 12). Together, these findings highlight the ongoing public health significance of ischemic HF, which represents the downstream clinical consequence of chronic coronary atherosclerosis, recurrent myocardial injury, and progressive ventricular remodeling [20, 21]. Given that CAD remains the leading cause of HF in many developed countries, understanding the evolving epidemiology of CAD‐related HF mortality is essential for guiding prevention strategies and optimizing cardiovascular care delivery.

The temporal pattern observed in this study suggests a multiphase epidemiologic transition in CAD‐HF mortality over the past two decades. Between 1999 and approximately 2012, mortality declined significantly across most demographic groups. This early decline parallels national improvements in cardiovascular outcomes observed in prior studies, where heart disease mortality decreased substantially during the early 2000s. These improvements are consistent with advances in cardiovascular prevention and treatment, including widespread adoption of statin therapy, improved hypertension control, smoking cessation initiatives, and rapid expansion of reperfusion therapy for AMI [22, 23, 24]. The increasing implementation of guideline‐directed medical therapy and improvements in emergency cardiovascular care systems also contributed to reductions in case fatality rates following myocardial infarction.

From 2012 through approximately 2018, mortality rates appeared to plateau across several demographic strata. Similar plateaus in cardiovascular mortality have been reported in national analyses during this period. This stabilization coincided with the rising prevalence of cardiometabolic risk factors, including obesity, diabetes mellitus, and metabolic syndrome, all of which are strongly associated with both CAD progression and the development of HF [25, 26]. For example, the prevalence of obesity in U.S. adults increased substantially during this period, contributing to increased metabolic stress and cardiovascular risk.

Between 2019 and 2021, CAD–HF mortality temporarily increased across demographic groups, reflecting reversals in previously improving trends [27, 28]. This coincided with the COVID‐19 pandemic, which caused direct cardiovascular complications, acute myocardial injury, myocarditis, thrombotic events, arrhythmias, and acute decompensated HF exacerbating underlying coronary disease [29]. Indirectly, healthcare disruptions occurred as patients delayed care due to viral exposure fears, routine follow‐up and chronic disease management were interrupted, and hospitals faced resource strain during surges [29]. These combined biological and systemic factors likely contributed to the temporary rise in mortality among individuals with coexisting CAD and HF. Notably, a sensitivity analysis excluding deaths with COVID‐19 (U07.1) as a contributing cause demonstrated that the 2018–2021 mortality spike persisted after removing COVID‐19‐associated co‐coded deaths (AAMR peak: 47.19 vs. 49.33 per 100,000 in the primary analysis), supporting the conclusion that this increase reflects true cardiovascular deterioration rather than a certification artifact.

Analyses by sex revealed that, while both men and women experienced significant declines in cardiovascular mortality over the study period, absolute mortality rates remained consistently higher among men. In contrast, the relative reduction was greater in women, reflecting a steeper decline over time. These patterns suggest ongoing sex disparities in the cardiovascular mortality burden, suggesting relatively greater improvements in awareness, diagnosis, and treatment of cardiovascular disease among women, which historically has been underrecognized.

Several biological and behavioral mechanisms may contribute to this disparity. Estrogen has been shown to exert cardioprotective effects through modulation of endothelial function, lipid metabolism, and inflammatory pathways involved in atherosclerosis [30, 31, 32]. This may explain why women tend to develop CAD later in life compared with men, delaying the onset of ischemic cardiomyopathy and HF. In addition, men have historically exhibited higher rates of traditional cardiovascular risk factors such as smoking and earlier development of obstructive coronary disease, which may partially explain the observed differences in mortality patterns.

However, because ICD‐10 code I50 captures all HF phenotypes without distinguishing heart failure with reduced ejection fraction (HFrEF) from heart failure with preserved ejection fraction (HFpEF), some deaths may have involved HFpEF occurring in the presence of coincidental rather than causative CAD. This limitation is particularly relevant to the observed sex and age‐specific trends, as HFpEF disproportionately affects older women.

Age‐related trends revealed some of the most striking findings of our study. Older adults experienced the highest mortality rates, reflecting the cumulative effects of long‐standing atherosclerosis, myocardial injury, and progressive cardiac remodeling. Age‐associated structural and functional cardiovascular changes, including increased arterial stiffness, reduced myocardial compliance, and impaired endothelial function, heighten vulnerability to CAD and HF. Comorbid conditions such as chronic kidney disease and diabetes further complicate management and increase mortality risk [33].

In contrast, middle‐aged adults demonstrated periods of increasing mortality in recent years, while young adults experienced a concerning rise beginning around 2010. Although absolute rates remain lower in younger populations, this trend is worrisome and warrants further investigation.

Rising obesity, diabetes, sedentary lifestyles, and substance use disorders may accelerate early cardiovascular disease. Socioeconomic stressors, barriers to healthcare access, and lower rates of routine screening may delay diagnosis and treatment of risk factors in these populations [34]. These findings suggest that the next generation may face a greater burden of chronic cardiovascular disease, underscoring the need for age‐targeted preventive strategies. However, because ICD‐10 code I50 captures all HF phenotypes without distinguishing HFrEF from HFpEF, some deaths may have involved HFpEF occurring in the presence of coincidental rather than causative CAD. This limitation is particularly relevant to the observed sex‐ and age‐specific trends, as HFpEF disproportionately affects older women.

Racial and ethnic disparities were also evident in our analysis. White adults had the highest overall AAMRs, followed by Black adults and other racial or ethnic groups. Although mortality declined across all racial and ethnic groups, the magnitude of improvement varied, reflecting persistent inequities in cardiovascular health outcomes in the United States. These disparities likely arise from complex interactions among biological risk factors, socioeconomic conditions, healthcare access, and structural determinants of health. Black populations have historically experienced a disproportionate burden of cardiovascular disease and its associated risk factors. Higher prevalence of hypertension, diabetes mellitus, obesity, and chronic kidney disease contributes to accelerated coronary atherosclerosis and earlier development of left ventricular dysfunction, increasing the likelihood of progression to ischemic cardiomyopathy and HF [35, 36, 37]. Hypertension, which is more prevalent and often more severe among Black adults, is a key contributor to CAD and adverse cardiac remodeling. Structural and socioeconomic determinants, including differences in healthcare access, insurance coverage, neighborhood environments, and preventive care availability, further influence these disparities and may lead to delayed diagnosis and suboptimal risk factor control [38]. Limited access to specialty cardiology services, cardiac rehabilitation, and guideline‐directed medical therapy may accelerate progression to chronic HF. Conversely, improvements observed among Hispanic and Asian populations may partly reflect the “healthy immigrant effect,” as well as cultural differences in diet, lifestyle, and community support structures [39]. Nonetheless, persistent disparities highlight the need for targeted public health strategies to improve cardiovascular risk factor control and equitable access to care.

Geographic disparities were pronounced across U.S. census regions. The Midwest and Southern regions demonstrated higher mortality rates compared with the Northeast and Western United States, consistent with previously described geographic clustering of cardiovascular disease commonly referred to as the “cardiovascular belt” [40]. These regions historically exhibit higher prevalence of hypertension, diabetes, smoking, and obesity.

Regional differences in mortality may also reflect variations in healthcare access, lower median income levels, public health infrastructure, and lifestyle behaviors, including dietary patterns and physical activity levels. These geographic patterns emphasize the importance of regionally targeted public health strategies aimed at reducing cardiovascular risk factors and improving access to cardiovascular care.

Consistent with prior epidemiologic research, rural populations exhibited persistently higher mortality rates compared with urban populations. Although mortality declined in both settings over time, improvements were greater in metropolitan areas, leading to persistent rural–urban disparities.

Several factors may contribute to these differences. Rural communities often face reduced access to specialized cardiovascular care, fewer tertiary care centers, longer travel distances for emergency services, and limited availability of cardiology specialists [41]. Socioeconomic barriers, including lower income levels and reduced healthcare coverage, may further exacerbate disparities in cardiovascular outcomes.

Substantial heterogeneity in mortality was observed across individual states. States such as West Virginia and Oklahoma demonstrated some of the highest mortality rates, whereas states including Hawaii and Alaska exhibited comparatively lower rates. These findings are consistent with broader state‐level differences in cardiovascular disease burden reported in national epidemiologic studies [41, 42].

State‐level variation likely reflects differences in population health profiles, prevalence of cardiovascular risk factors, healthcare infrastructure and regional socioeconomic conditions [42]. Public health initiatives at the state level may therefore play a critical role in addressing localized disparities in cardiovascular mortality.

Analysis of death locations demonstrated that the largest proportion of deaths occurred in inpatient medical facilities, followed by deaths occurring at home and in nursing homes or long‐term care facilities. Smaller proportions occurred in hospice facilities and outpatient or emergency departments.

These findings highlight the complex care needs of patients with advanced cardiovascular disease and emphasize the importance of outpatient HF management, coordinated care transitions, and palliative care services for patients with advanced disease [21]. Expanding access to community‐based HF management programs may help reduce hospitalizations and improve quality of life for patients with advanced CAD‐related HF.

Subgroup analyses comparing AMI–related ischemic heart disease and chronic ischemic cardiomyopathy demonstrated distinct epidemiologic patterns. Mortality associated with AMI declined more rapidly over time, likely reflecting substantial improvements in reperfusion therapy, early diagnosis, and modern management of acute coronary syndromes [23]. Advances such as primary percutaneous coronary intervention and improved pharmacologic therapies have significantly reduced short‐term mortality following myocardial infarction.

In contrast, chronic ischemic cardiomyopathy continues to represent a substantial contributor to HF mortality [43]. As survival following myocardial infarction improves, more patients live long enough to develop progressive ventricular dysfunction and chronic HF, which may partially explain the sustained burden of CAD‐related HF mortality observed in this analysis [44].

### 6.1 Healthcare Implications

Although overall mortality declined during the study period, the slowing pace of improvement and the recent periods of increasing mortality signal a potential turning point in cardiovascular epidemiology. Without targeted interventions addressing cardiometabolic disease and healthcare disparities, the burden of ischemic HF may continue to rise in the coming decades. Future research should focus on identifying high‐risk populations, improving long‐term management of CAD survivors, and developing strategies to mitigate the progression from acute coronary disease to chronic HF. In parallel, greater emphasis on optimization and equitable implementation of guideline‐directed medical therapy is needed, alongside the integration of emerging pharmacologic agents and evidence‐based device therapies to improve outcomes. Strengthening multidisciplinary care models, enhancing treatment adherence, and leveraging digital health tools for early detection and monitoring may further support effective management of patients at risk for ischemic HF.

### 6.2 Limitations

This study has several limitations that should be considered when interpreting the findings. First, mortality data derived from death certificates may be subject to misclassification or reporting inaccuracies, particularly regarding contributing causes of death. The use of ICD‐10 code I50 does not distinguish between HF subtypes (HFrEF vs. HFpEF), potentially introducing misclassification, particularly among older adults and women, where HFpEF is more prevalent. Inclusion of ICD‐10 code I11.0 may capture non‐ischemic HF; however, this potential misclassification is unlikely to substantially affect the study's conclusions. Second, the CDC WONDER database does not provide detailed clinical information such as the severity of CAD, HF phenotype, treatment history, or comorbid conditions, which may influence outcomes. Thirdly, this study represents an observational ecological analysis, and causal relationships between demographic factors and mortality trends cannot be definitively established; therefore, mechanistic explanations offered in the Discussion are hypothesis‐generating and should be interpreted in the context of supporting literature rather than as conclusions from the present data. In addition, variations in death certificate coding practices across states and over time may introduce measurement variability. Furthermore, CAD or HF may not always be listed as contributing causes on death certificates, potentially leading to underestimation of the true burden of coexisting disease. Finally, because analyses were conducted at the population level, individual‐level risk factors and healthcare access patterns could not be directly evaluated.

## 7 Conclusion

This nationwide analysis spanning more than two decades demonstrates that mortality involving coexisting CAD and HF remains a substantial and evolving public health challenge in the United States. Although mortality has declined significantly over time, the overall burden remains unacceptably high. Early reductions in mortality likely reflected major advances in cardiovascular prevention and treatment, with subsequent stabilization and episodic increases highlighting the growing influence of cardiometabolic risk factors and persistent healthcare disparities. Marked differences across sex, race and ethnicity, geographic region, and urbanization status emphasize the need for targeted public health interventions and equitable access to cardiovascular care. Continued progress in preventive cardiology, early detection of CAD, and optimization of HF management will be essential to reducing the long‐term burden of ischemic HF.

## Author Contributions

**Sahil Bhagia:** conceptualization of the study, supervising project workflow, project administration, drafting and revising the manuscript. **Muhammad Salman Mustafa:** conceptualization, data curation, checking validation of data, drafting and reviewing manuscript sections. **Daniyal Khalid:** literature review, manuscript writing, and assisting with data interpretation. **Zainab Mazhar Baig:** methodology, data investigation, manuscript writing, and reviewing. **Noor Ahmed:** data visualization, reviewing results, critical manuscript revisions. **Muhammad Mussawir Khuhro:** data analysis and interpretation, proofreading final manuscript. **Hasan Nazeer Khan:** data visualization and drafting the manuscript. **Duaa Mubashir:** assistance in project administration and writing the manuscript. **Sara Arsalan:** assistance with data curation and formatting the final manuscript. **Muhammad Bilal Khan:** methodology and manuscript writing. **Hasibullah Aminpoor:** project administration and reviewing the final manuscript.

## Funding

The authors have nothing to report.

## Ethics Statement

The authors have nothing to report.

## Consent

The authors have nothing to report.

## Conflicts of Interest

The authors declare no conflicts of interest.

## Transparency Statement

The lead author (Sahil Bhagia) affirms that this manuscript is an honest, accurate, and transparent account of the study being reported. No important aspects of the study have been omitted, and any discrepancies from the study as originally planned have been clearly explained.

## Supporting information


**Table S1:** Demographics of Coronary Artery Disease (CAD) and Heart Failure (HF) in adults in the US, 1999–2024, by Overall, Sex, Age groups and Race. **Table S2:** Demographics of Coronary Artery Disease (CAD) and Heart Failure (HF) in adults in the US, 1999–2024, by Place of Deaths. **Table S3:** Annual Percentage Change (APC) for Coronary Artery Disease (CAD) and Heart Failure (HF)‐related mortality in U.S. Adults, 1999–2024. **Table S4:** Age‐adjusted mortality rates of Coronary Artery Disease (CAD) and Heart Failure (HF) in adults in the US, 1999–2024, by Overall and Sex. **Table S5:** Age‐adjusted mortality rates of Coronary Artery Disease (CAD) and Heart Failure (HF) in adults in the US, 1999–2024, by Age groups. **Table S6:** Age‐adjusted mortality rates of Coronary Artery Disease (CAD) and Heart Failure (HF) in adults in the US, 1999–2024, by Race. **Table S7:** Age‐adjusted mortality rates of Coronary Artery Disease (CAD) and Heart Failure (HF) in adults in the US, 1999–2024, by Census region. **Table S8:** Age‐adjusted mortality rates of Coronary Artery Disease (CAD) and Heart Failure (HF) in adults in the US, 1999–2020, by Urbanization. **Table S9:** Age‐adjusted mortality rates of Coronary Artery Disease (CAD) and Heart Failure (HF) in adults in the US, 1999–2020 and 2021–2024, by States. **Table S10:** Age‐adjusted mortality rates of Coronary Artery Disease (CAD) and Heart Failure (HF) in adults in the US, 1999–2024, by Clinical Presentation. **Table S11:** Age‐adjusted mortality rates of Coronary Artery Disease (CAD) and Heart Failure (HF) in adults in the US, 2018–2024, by sensitivity analysis with COVID‐19 deaths vs without COVID‐19 deaths. **Table S12:** Age‐adjusted mortality rates of Coronary Artery Disease (CAD) and Heart Failure (HF) in adults in the US, 1999–2024, by sensitivity analysis without hypertensive HF vs with hypertensive HF.

## Data Availability

The data that support the findings of this study are available in CDC WONDER at https://wonder.cdc.gov/mcd.html. These data were derived from the following resources available in the public domain: Multiple Cause of Death, 1999–2020, https://wonder.cdc.gov/mcd-icd10.html.
