# Supplementary material for: Trends and Racial‐Geographic Disparities in Coexisting Coronary Artery Disease (CAD) and Heart Failure (HF) Related Mortality Among U.S. Adults, 1999–2024
Source: Health Sci Rep. 2026 Jun 9;9(6):e72635. doi: 10.1002/hsr2.72635 (PMC13250392; doi:10.1002/hsr2.72635)
Supplement: Supplementary file 1 — Table S1: Demographics of Coronary Artery Disease (CAD) and Heart Failure (HF) in adults in the US, 1999–2024, by Overall, Sex, Age groups and Race. Table S2: Demographics of Coronary Artery Disease (CAD) and Heart Failure (HF) in adults in the US, 1999–2024, by Place of Deaths. Table S3: Annual Percentage Change (APC) for Coronary Artery Disease (CAD) and Heart Failure (HF)‐related mortality in U.S. Adults, 1999–2024. Table S4: Age‐adjusted mortality rates of Coronary Artery Disease (CAD) and Heart Failure (HF) in adults in the US, 1999–2024, by Overall and Sex. Table S5: Age‐adjusted mortality rates of Coronary Artery Disease (CAD) and Heart Failure (HF) in adults in the US, 1999–2024, by Age groups. Table S6: Age‐adjusted mortality rates of Coronary Artery Disease (CAD) and Heart Failure (HF) in adults in the US, 1999–2024, by Race. Table S7: Age‐adjusted mortality rates of Coronary Artery Disease (CAD) and Heart Failure (HF) in adults in the US, 1999–2024, by Census region. Table S8: Age‐adjusted mortality rates of Coronary Artery Disease (CAD) and Heart Failure (HF) in adults in the US, 1999–2020, by Urbanization. Table S9: Age‐adjusted mortality rates of Coronary Artery Disease (CAD) and Heart Failure (HF) in adults in the US, 1999–2020 and 2021–2024, by States. Table S10: Age‐adjusted mortality rates of Coronary Artery Disease (CAD) and Heart Failure (HF) in adults in the US, 1999–2024, by Clinical Presentation. Table S11: Age‐adjusted mortality rates of Coronary Artery Disease (CAD) and Heart Failure (HF) in adults in the US, 2018–2024, by sensitivity analysis with COVID‐19 deaths vs without COVID‐19 deaths. Table S12: Age‐adjusted mortality rates of Coronary Artery Disease (CAD) and Heart Failure (HF) in adults in the US, 1999–2024, by sensitivity analysis without hypertensive HF vs with hypertensive HF. [file HSR2-9-e72635-s001.docx]

**Tables:**

**SUPPLEMENTARY TABLE 1:** Demographics of Coronary Artery Disease (CAD) and Heart Failure (HF) in adults in the US, 1999–2024, by Overall, Sex, Age groups and Race

**SUPPLEMENTARY TABLE 2:** Demographics of Coronary Artery Disease (CAD) and Heart Failure (HF) in adults in the US, 1999–2024, by Place of Deaths.

**SUPPLEMENTARY TABLE 3:** Annual Percentage Change (APC) for Coronary Artery Disease (CAD) and Heart Failure (HF)-related mortality in U.S. Adults; 1999-2024

**SUPPLEMENTARY TABLE 4:** Age-adjusted mortality rates of Coronary Artery Disease (CAD) and Heart Failure (HF) in adults in the US, 1999–2024, by Overall and Sex

**SUPPLEMENTARY TABLE 5:** Age-adjusted mortality rates of Coronary Artery Disease (CAD) and Heart Failure (HF) in adults in the US, 1999–2024, by Age groups

**SUPPLEMENTARY TABLE 6:** Age-adjusted mortality rates of Coronary Artery Disease (CAD) and Heart Failure (HF) in adults in the US, 1999–2024, by Race

**SUPPLEMENTARY TABLE 7:** Age-adjusted mortality rates of Coronary Artery Disease (CAD) and Heart Failure (HF) in adults in the US, 1999–2024, by Census region

**SUPPLEMENTARY TABLE 8:** Age-adjusted mortality rates of Coronary Artery Disease (CAD) and Heart Failure (HF) in adults in the US, 1999–2020, by Urbanization

**SUPPLEMENTARY TABLE 9:** Age-adjusted mortality rates of Coronary Artery Disease (CAD) and Heart Failure (HF) in adults in the US, 1999–2020 and 2021-2024, by States

**SUPPLEMENTARY TABLE 10:** Age-adjusted mortality rates of Coronary Artery Disease (CAD) and Heart Failure (HF) in adults in the US, 1999–2024, by Clinical Presentation

**SUPPLEMENTARY TABLE 11:** Age-adjusted mortality rates of Coronary Artery Disease (CAD) and Heart Failure (HF) in adults in the US, 2018-2024, by sensitivity analysis With Covid deaths VS without Covid deaths.

**SUPPLEMENTARY TABLE 12:** Age-adjusted mortality rates of Coronary Artery Disease (CAD) and Heart Failure (HF) in adults in the US, 1999–2024, by sensitivity analysis without hypertensive HF VS with Hypertensive HF.

| **Demographics** | | | | | | | | | | | | |
| --- | --- | --- | --- | --- | --- | --- | --- | --- | --- | --- | --- | --- |
| **Year** | **Overall** | **Female** | **Male** | **Young adults** | **Middle Age Adults** | **Old adults** | **American Indian or Alaska Native** | **Asian or Pacific Islander** | **Black or African American** | **White** | **Hispanic or Latino** | **Population** |
| **1999** | **125702** | **68762** | **56940** | **453** | **7873** | **117376** | **372** | **1286** | **8656** | **115388** | **3998** | **180408769** |
| **2000** | **124675** | **67846** | **56829** | **478** | **8103** | **116094** | **369** | **1218** | **8864** | **114224** | **4045** | **181984640** |
| **2001** | **121705** | **66139** | **55566** | **442** | **7914** | **113349** | **331** | **1315** | **8389** | **111670** | **4233** | **184305128** |
| **2002** | **119272** | **63980** | **55292** | **520** | **8076** | **110676** | **415** | **1335** | **8467** | **109055** | **4206** | **186208028** |
| **2003** | **117843** | **62826** | **55017** | **453** | **8271** | **109119** | **461** | **1406** | **8288** | **107688** | **4329** | **188090429** |
| **2004** | **113681** | **59518** | **54163** | **481** | **8084** | **105116** | **457** | **1434** | **8051** | **103739** | **4237** | **190205384** |
| **2005** | **115591** | **60321** | **55270** | **461** | **8386** | **106744** | **399** | **1558** | **8488** | **105146** | **4545** | **192551384** |
| **2006** | **109695** | **56306** | **53389** | **477** | **8163** | **101055** | **460** | **1604** | **8026** | **99605** | **4559** | **195019359** |
| **2007** | **105326** | **53721** | **51605** | **463** | **7963** | **96900** | **417** | **1598** | **7812** | **95499** | **4478** | **197403777** |
| **2008** | **105161** | **53042** | **52119** | **422** | **8091** | **96648** | **432** | **1577** | **7724** | **95428** | **4529** | **199795090** |
| **2009** | **100099** | **49317** | **50782** | **384** | **7927** | **91788** | **450** | **1638** | **7498** | **90513** | **4416** | **202107016** |
| **2010** | **99841** | **48642** | **51199** | **363** | **7871** | **91607** | **470** | **1610** | **7569** | **90192** | **4526** | **203891983** |
| **2011** | **99497** | **47776** | **51721** | **449** | **8021** | **91027** | **485** | **1680** | **7521** | **89811** | **4696** | **206592936** |
| **2012** | **98567** | **46548** | **52019** | **409** | **8208** | **89950** | **499** | **1772** | **7674** | **88622** | **4827** | **208826037** |
| **2013** | **100281** | **46499** | **53782** | **444** | **8303** | **91534** | **520** | **1942** | **7985** | **89834** | **5172** | **211085314** |
| **2014** | **101135** | **45922** | **55213** | **443** | **8686** | **92006** | **585** | **1912** | **8288** | **90350** | **5424** | **213809280** |
| **2015** | **104569** | **47452** | **57117** | **443** | **9200** | **94926** | **629** | **2202** | **8885** | **92853** | **5858** | **216553817** |
| **2016** | **104706** | **45979** | **58727** | **473** | **9635** | **94598** | **665** | **2144** | **8926** | **92971** | **6067** | **218641417** |
| **2017** | **108800** | **47307** | **61493** | **476** | **9980** | **98344** | **637** | **2456** | **9518** | **96189** | **6473** | **221447331** |
| **2018** | **111801** | **47839** | **63962** | **517** | **10414** | **100870** | **742** | **2617** | **9711** | **98731** | **6822** | **223311190** |
| **2019** | **114741** | **48431** | **66310** | **550** | **10665** | **103526** | **711** | **2659** | **10345** | **101026** | **7129** | **224981167** |
| **2020** | **126266** | **52526** | **73740** | **667** | **12476** | **113123** | **885** | **3135** | **12381** | **109865** | **8511** | **226635013** |
| **2021** | **127488** | **52198** | **75290** | **760** | **13103** | **113625** | **798** | **2849** | **12093** | **110941** | **8649** | **228238412** |
| **2022** | **126591** | **51970** | **74621** | **742** | **12241** | **113608** | **736** | **2972** | **11784** | **110309** | **8175** | **229508599** |
| **2023** | **123716** | **50169** | **73547** | **701** | **11934** | **111081** | **761** | **2838** | **11817** | **107495** | **8151** | **231529762** |
| **2024** | **123818** | **49379** | **74439** | **673** | **11736** | **111409** | **811** | **2987** | **12012** | **107160** | **8158** | **235615087** |
| **Totals** | **2930567** | **1390415** | **1540152** | **13144** | **241324** | **2676099** | **14497** | **51744** | **236772** | **2624304** | **146213** | **5398746349** |

**SUPPLEMENTARY TABLE 1:** Demographics of Coronary Artery Disease (CAD) and Heart Failure (HF) in adults in the US, 1999–2024, by Overall, Sex, Age groups and Race

| **Place of Death** | **No. of Deaths** | **% of Deaths** |
| --- | --- | --- |
| **Medical Facility - Inpatient** | **961698** | **32.80%** |
| **Medical Facility - Outpatient or ER** | **185468** | **6.30%** |
| **Medical Facility - Dead on Arrival** | **13780** | **0.47%** |
| **Medical Facility - Status unknown** | **2002** | **0.07%** |
| **Decedent's home** | **784100** | **26.80%** |
| **Hospice facility** | **124106** | **4.20%** |
| **Nursing home/long term care** | **742822** | **25.40%** |
| **Other** | **111480** | **3.80%** |
| **Place of death unknown** | **5111** | **0.17%** |
| **Total** | **2930567** | **100%** |
|  |  |  |

**SUPPLEMENTARY TABLE 2: Demographics of Coronary Artery Disease (CAD) and Heart Failure (HF) in adults in the US, 1999–2024, by Place of Deaths**

| **Annual and Average Annual Percentage Change (AAPC) + 95% Confidence Interval** | | | | | | |
| --- | --- | --- | --- | --- | --- | --- |
| **Overall and Sex** | | | | | | |
| **Variable** | **Lower Endpoint** | **Upper Endpoint** | **APC** | **Lower CI** | **Upper CI** | **P Value** |
| **Overall** | **1999** | **2012** | **-3.9665*** | **-4.2055** | **-3.727** | **< 0.000001** |
| **Overall** | **2012** | **2018** | **-0.314** | **-1.4228** | **0.8072** | **0.55788** |
| **Overall** | **2018** | **2021** | **4.7195*** | **0.0136** | **9.6469** | **0.049407** |
| **Overall** | **2021** | **2024** | **-3.6648*** | **-5.8537** | **-1.4251** | **0.003481** |
| **Female** | **1999** | **2005** | **-3.8461*** | **-4.44** | **-3.2486** | **< 0.000001** |
| **Female** | **2005** | **2011** | **-5.7009*** | **-6.57** | **-4.8237** | **< 0.000001** |
| **Female** | **2011** | **2018** | **-1.6263*** | **-2.3676** | **-0.8793** | **0.000494** |
| **Female** | **2018** | **2021** | **4.247** | **-0.0799** | **8.7612** | **0.053808** |
| **Female** | **2021** | **2024** | **-4.1139*** | **-6.0975** | **-2.0885** | **0.000898** |
| **Male** | **1999** | **2012** | **-3.2988*** | **-3.5093** | **-3.0878** | **< 0.000001** |
| **Male** | **2012** | **2018** | **0.3374** | **-0.5579** | **1.2408** | **0.435634** |
| **Male** | **2018** | **2021** | **5.0498*** | **1.3209** | **8.9158** | **0.010876** |
| **Male** | **2021** | **2024** | **-3.5954*** | **-5.2661** | **-1.8953** | **0.000454** |
| **Overall** | **Full Range** | | **-2.0534*** | **-2.6685** | **-1.4344** | **< 0.000001** |
| **Female** | **Full Range** | | **-2.7749*** | **-3.3521** | **-2.1943** | **< 0.000001** |
| **Male** | **Full Range** | | **-1.5003*** | **-1.9879** | **-1.0103** | **< 0.000001** |
| **Age Group** | | | | | | |
| **Young Adults** | **1999** | **2006** | **2.0591** | **-1.6346** | **5.8915** | **0.256954** |
| **Young Adults** | **2006** | **2010** | **-7.1522** | **-18.329** | **5.5541** | **0.23648** |
| **Young Adults** | **2010** | **2022** | **5.8112*** | **4.1196** | **7.5302** | **0.000002** |
| **Young Adults** | **2022** | **2024** | **-6.2279** | **-23.2303** | **14.54** | **0.503732** |
| **Middle Age Adults** | **1999** | **2011** | **-3.5189*** | **-3.7692** | **-3.268** | **< 0.000001** |
| **Middle Age Adults** | **2011** | **2018** | **2.9644*** | **2.2249** | **3.7093** | **< 0.000001** |
| **Middle Age Adults** | **2018** | **2021** | **8.3756*** | **4.3967** | **12.5062** | **0.000359** |
| **Middle Age Adults** | **2021** | **2024** | **-3.2231*** | **-5.0053** | **-1.4075** | **0.001903** |
| **Older Adults** | **1999** | **2012** | **-4.0344*** | **-4.2838** | **-3.7844** | **< 0.000001** |
| **Older Adults** | **2012** | **2018** | **-0.6833** | **-1.8339** | **0.4807** | **0.228944** |
| **Older Adults** | **2018** | **2021** | **4.3299** | **-0.6317** | **9.5393** | **0.083472** |
| **Older Adults** | **2021** | **2024** | **-3.6989*** | **-5.9583** | **-1.3852** | **0.004093** |
| **Young Adults** | **Full Range** | | **1.5928** | **-1.0847** | **4.3428** | **0.246205** |
| **Middle Age Adults** | **Full Range** | | **-0.329** | **-0.8359** | **0.1806** | **0.205321** |
| **Older Adults** | **Full Range** | | **-2.2245*** | **-2.8704** | **-1.5743** | **< 0.000001** |
| **Race/Ethnicity** | | | | | | |
| **American Indian or Alaska Native** | **1999** | **2024** | **-1.5762*** | **-2.0031** | **-1.1474** | **< 0.000001** |
| **Asian or Pacific Islander** | **1999** | **2014** | **-3.8227*** | **-4.3099** | **-3.333** | **< 0.000001** |
| **Asian or Pacific Islander** | **2014** | **2021** | **1.3027** | **-0.2492** | **2.8787** | **0.095147** |
| **Asian or Pacific Islander** | **2021** | **2024** | **-4.9278*** | **-8.8188** | **-0.8707** | **0.020498** |
| **Black or African American** | **1999** | **2012** | **-3.7265*** | **-4.0799** | **-3.3717** | **< 0.000001** |
| **Black or African American** | **2012** | **2018** | **0.3435** | **-1.1766** | **1.887** | **0.638957** |
| **Black or African American** | **2018** | **2021** | **6.2001*** | **0.0742** | **12.701** | **0.047541** |
| **Black or African American** | **2021** | **2024** | **-3.9466*** | **-6.6992** | **-1.1127** | **0.009899** |
| **White** | **1999** | **2012** | **-3.9347*** | **-4.1766** | **-3.6922** | **< 0.000001** |
| **White** | **2012** | **2018** | **-0.2458** | **-1.3489** | **0.8697** | **0.643937** |
| **White** | **2018** | **2021** | **4.8965*** | **0.0431** | **9.9853** | **0.048198** |
| **White** | **2021** | **2024** | **-3.3441*** | **-5.5798** | **-1.0554** | **0.00735** |
| **Hispanic or Latino** | **1999** | **2012** | **-4.2519*** | **-4.5684** | **-3.9343** | **< 0.000001** |
| **Hispanic or Latino** | **2012** | **2018** | **-0.374** | **-1.5772** | **0.8439** | **0.520978** |
| **Hispanic or Latino** | **2018** | **2021** | **5.0641*** | **0.2096** | **10.1536** | **0.041783** |
| **Hispanic or Latino** | **2021** | **2024** | **-7.0080*** | **-9.1174** | **-4.8497** | **0.000007** |
| **American Indian or Alaska Native** | **Full Range** | | **-1.5762*** | **-2.0031** | **-1.1474** | **< 0.000001** |
| **Asian or Pacific Islander** | **Full Range** | | **-2.5496*** | **-3.2099** | **-1.8847** | **< 0.000001** |
| **Black or African American** | **Full Range** | | **-1.6402*** | **-2.4463** | **-0.8275** | **0.000082** |
| **White** | **Full Range** | | **-1.9615*** | **-2.5908** | **-1.3281** | **< 0.000001** |
| **Hispanic or Latino** | **Full Range** | | **-2.5941*** | **-3.2334** | **-1.9506** | **< 0.000001** |
| **Census Region** | | | | | | |
| **Northeast** | **1999** | **2005** | **-3.4936*** | **-4.1223** | **-2.8609** | **< 0.000001** |
| **Northeast** | **2005** | **2009** | **-5.7718*** | **-7.7287** | **-3.7734** | **0.000048** |
| **Northeast** | **2009** | **2017** | **-2.1012*** | **-2.6877** | **-1.5111** | **0.000006** |
| **Northeast** | **2017** | **2021** | **1.398** | **-0.7851** | **3.6292** | **0.189832** |
| **Northeast** | **2021** | **2024** | **-4.8603*** | **-6.9911** | **-2.6806** | **0.000439** |
| **Midwest** | **1999** | **2012** | **-3.9284*** | **-4.1821** | **-3.6741** | **< 0.000001** |
| **Midwest** | **2012** | **2018** | **0.0342** | **-1.1669** | **1.25** | **0.952663** |
| **Midwest** | **2018** | **2021** | **5.3337*** | **0.1361** | **10.801** | **0.044848** |
| **Midwest** | **2021** | **2024** | **-2.7836*** | **-5.1593** | **-0.3485** | **0.028001** |
| **South** | **1999** | **2005** | **-3.2234*** | **-3.7304** | **-2.7136** | **< 0.000001** |
| **South** | **2005** | **2011** | **-4.9429*** | **-5.6421** | **-4.2386** | **< 0.000001** |
| **South** | **2011** | **2018** | **0.0482** | **-0.5046** | **0.6042** | **0.8528** |
| **South** | **2018** | **2021** | **5.9994*** | **2.8933** | **9.1994** | **0.001091** |
| **South** | **2021** | **2024** | **-3.1372*** | **-4.4908** | **-1.7644** | **0.000345** |
| **West** | **1999** | **2003** | **-2.7351*** | **-3.9009** | **-1.555** | **0.000304** |
| **West** | **2003** | **2012** | **-4.1614*** | **-4.5755** | **-3.7456** | **< 0.000001** |
| **West** | **2012** | **2018** | **-0.5292** | **-1.4022** | **0.3515** | **0.214207** |
| **West** | **2018** | **2021** | **4.2166*** | **0.4667** | **8.1064** | **0.030277** |
| **West** | **2021** | **2024** | **-4.6439*** | **-6.335** | **-2.9223** | **0.000086** |
| **Northeast** | **Full Range** | | **-2.8195*** | **-3.3494** | **-2.2866** | **< 0.000001** |
| **Midwest** | **Full Range** | | **-1.7750*** | **-2.4485** | **-1.0969** | **< 0.000001** |
| **South** | **Full Range** | | **-1.6589*** | **-2.0797** | **-1.2363** | **< 0.000001** |
| **West** | **Full Range** | | **-2.1527*** | **-2.6689** | **-1.6338** | **< 0.000001** |
| **Urbanization** | | | | | | |
| **Urban** | **1999** | **2005** | **-3.4414*** | **-3.9379** | **-2.9423** | **< 0.000001** |
| **Urban** | **2005** | **2009** | **-5.2993*** | **-6.8183** | **-3.7555** | **0.000054** |
| **Urban** | **2009** | **2012** | **-3.0049** | **-6.186** | **0.2841** | **0.067892** |
| **Urban** | **2012** | **2018** | **-0.4905** | **-1.223** | **0.2475** | **0.163454** |
| **Urban** | **2018** | **2020** | **4.3885*** | **1.2102** | **7.6667** | **0.012553** |
| **Rural** | **1999** | **2013** | **-3.2940*** | **-3.547** | **-3.0402** | **< 0.000001** |
| **Rural** | **2013** | **2018** | **0.7106** | **-1.1368** | **2.5925** | **0.425805** |
| **Rural** | **2018** | **2020** | **4.9656** | **-0.6115** | **10.8557** | **0.077692** |
| **Urban** | **Full Range** | | **-2.1808*** | **-2.752** | **-1.6063** | **< 0.000001** |
| **Rural** | **Full Range** | | **-1.5900*** | **-2.2208** | **-0.9552** | **0.000001** |
| **Chronic vs Acute** | | | | | | |
| **Chronic Ischemic Cardiomyopathy** | **1999** | **2013** | **-2.3893*** | **-2.7623** | **-2.0149** | **< 0.000001** |
| **Chronic Ischemic Cardiomyopathy** | **2013** | **2021** | **1.6691*** | **0.6065** | **2.7429** | **0.003893** |
| **Chronic Ischemic Cardiomyopathy** | **2021** | **2024** | **-3.7384** | **-7.4702** | **0.1438** | **0.058011** |
| **Acute MI** | **1999** | **2002** | **-3.5412*** | **-5.2673** | **-1.7836** | **0.000944** |
| **Acute MI** | **2002** | **2011** | **-5.8645*** | **-6.2761** | **-5.451** | **< 0.000001** |
| **Acute MI** | **2011** | **2018** | **-0.8196*** | **-1.5743** | **-0.0592** | **0.036865** |
| **Acute MI** | **2018** | **2021** | **5.7041*** | **1.5099** | **10.0716** | **0.011374** |
| **Acute MI** | **2021** | **2024** | **-4.8779*** | **-6.7584** | **-2.9594** | **0.000146** |
| **Chronic Ischemic Cardiomyopathy** | **Full Range** | | **-1.2736*** | **-1.8428** | **-0.7011** | **0.000014** |
| **Acute MI** | **Full Range** | | **-2.7350*** | **-3.2944** | **-2.1725** | **< 0.000001** |

**SUPPLEMENTARY TABLE 3:** Annual Percentage Change (APC) for Coronary Artery Disease (CAD) and Heart Failure (HF)-related mortality in U.S. Adults; 1999-2024

| **Age-Adjusted Rate (95% CI)** | | | |
| --- | --- | --- | --- |
| **Year** | **Overall** | **Female** | **Male** |
| **1999** | **71.62  (71.22 to 72.01)** | **60.64 (60.18 to 61.1)** | **88.4 (87.66 to 89.14)** |
| **2000** | **70.03  (69.64 to 70.41)** | **59.1 (58.65 to 59.54)** | **86.85 (86.12 to 87.58)** |
| **2001** | **67.28  (66.90 to 67.66)** | **56.88 (56.44 to 57.31)** | **82.98 (82.27 to 83.68)** |
| **2002** | **65.03  (64.66 to 65.40)** | **54.48 (54.05 to 54.9)** | **81.14 (80.45 to 81.83)** |
| **2003** | **63.14  (62.78 to 63.50)** | **52.79 (52.37 to 53.2)** | **78.96 (78.29 to 79.63)** |
| **2004** | **60.08  (59.73 to 60.43)** | **49.53 (49.13 to 49.93)** | **76.14 (75.49 to 76.79)** |
| **2005** | **59.78  (59.44 to 60.13)** | **49.34 (48.95 to 49.74)** | **75.88 (75.24 to 76.52)** |
| **2006** | **55.52  (55.19 to 55.85)** | **45.15 (44.78 to 45.53)** | **71.21 (70.6 to 71.83)** |
| **2007** | **52.13  (51.82 to 52.45)** | **42.26 (41.89 to 42.62)** | **67.01 (66.43 to 67.6)** |
| **2008** | **50.91  (50.60 to 51.22)** | **40.94 (40.59 to 41.3)** | **65.88 (65.31 to 66.46)** |
| **2009** | **47.49  (47.19 to 47.78)** | **37.45 (37.12 to 37.79)** | **62.52 (61.97 to 63.07)** |
| **2010** | **46.53  (46.24 to 46.82)** | **36.39 (36.07 to 36.72)** | **61.8 (61.26 to 62.34)** |
| **2011** | **45.03  (44.75 to 45.32)** | **34.87 (34.55 to 35.19)** | **60 (59.47 to 60.52)** |
| **2012** | **43.49  (43.22 to 43.76)** | **33.32 (33.01 to 33.63)** | **58.39 (57.88 to 58.9)** |
| **2013** | **43.20  (42.93 to 43.47)** | **32.67 (32.37 to 32.98)** | **58.47 (57.97 to 58.97)** |
| **2014** | **42.56  (42.30 to 42.83)** | **31.72 (31.42 to 32.01)** | **58.11 (57.62 to 58.6)** |
| **2015** | **43.06  (42.80 to 43.33)** | **32.3 (32 to 32.59)** | **58.36 (57.87 to 58.84)** |
| **2016** | **42.25  (41.99 to 42.51)** | **30.8 (30.52 to 31.09)** | **58.36 (57.89 to 58.84)** |
| **2017** | **42.86  (42.61 to 43.12)** | **31.1 (30.81 to 31.38)** | **59.31 (58.83 to 59.79)** |
| **2018** | **43.06  (42.81 to 43.32)** | **30.92 (30.64 to 31.2)** | **59.94 (59.47 to 60.41)** |
| **2019** | **43.31  (43.05 to 43.56)** | **30.84 (30.56 to 31.12)** | **60.43 (59.96 to 60.9)** |
| **2020** | **46.81  (46.55 to 47.07)** | **33.12 (32.83 to 33.4)** | **65.56 (65.09 to 66.04)** |
| **2021** | **49.33  (49.06 to 49.61)** | **34.81 (34.51 to 35.11)** | **69.07 (68.57 to 69.58)** |
| **2022** | **46.26  (46.01 to 46.52)** | **32.34 (32.06 to 32.62)** | **65.69 (65.21 to 66.17)** |
| **2023** | **45.28  (45.03 to 45.54)** | **31.78 (31.5 to 32.06)** | **63.52 (63.05 to 63.99)** |
| **2024** | **43.74  (43.50 to 43.99)** | **30.33 (30.06 to 30.6)** | **61.56 (61.11 to 62.01)** |
| **Overall** | **51.14  (50.84 to 51.44)** | **39.84 (39.50 to 40.17)** | **67.52 (66.96 to 68.07)** |

**SUPPLEMENTARY TABLE 4:** Age-adjusted mortality rates of Coronary Artery Disease (CAD) and Heart Failure (HF) in adults in the US, 1999–2024, by Overall and Sex

| **Age-Adjusted Rate (95% CI)** | | | |
| --- | --- | --- | --- |
| **Year** | **Young Adults** | **Middle Age Adults** | **Older Adults** |
| **1999** | **0.54 (0.49 to 0.59)** | **13.04 (12.75 to 13.32)** | **342.26 (340.3 to 344.22)** |
| **2000** | **0.54 (0.49 to 0.58)** | **13.09 (12.81 to 13.38)** | **334.02 (332.1 to 335.94)** |
| **2001** | **0.53 (0.48 to 0.58)** | **12.36 (12.09 to 12.64)** | **321.26 (319.39 to 323.13)** |
| **2002** | **0.64 (0.58 to 0.69)** | **11.99 (11.73 to 12.25)** | **310.18 (308.35 to 312)** |
| **2003** | **0.54 (0.49 to 0.59)** | **11.75 (11.5 to 12.01)** | **301.15 (299.36 to 302.93)** |
| **2004** | **0.64 (0.58 to 0.69)** | **11.06 (10.82 to 11.31)** | **286.47 (284.74 to 288.2)** |
| **2005** | **0.54 (0.49 to 0.59)** | **11.07 (10.83 to 11.31)** | **285.17 (283.46 to 286.88)** |
| **2006** | **0.64 (0.58 to 0.69)** | **10.36 (10.13 to 10.58)** | **264.39 (262.76 to 266.02)** |
| **2007** | **0.58 (0.53 to 0.63)** | **9.82 (9.61 to 10.04)** | **248.1 (246.54 to 249.66)** |
| **2008** | **0.53 (0.48 to 0.58)** | **9.7 (9.49 to 9.92)** | **242.19 (240.66 to 243.72)** |
| **2009** | **0.48 (0.43 to 0.53)** | **9.29 (9.08 to 9.49)** | **225.5 (224.04 to 226.97)** |
| **2010** | **0.43 (0.38 to 0.47)** | **8.97 (8.77 to 9.17)** | **221.3 (219.86 to 222.74)** |
| **2011** | **0.59 (0.54 to 0.65)** | **8.91 (8.72 to 9.11)** | **213.36 (211.97 to 214.75)** |
| **2012** | **0.53 (0.48 to 0.58)** | **8.99 (8.8 to 9.19)** | **205.47 (204.12 to 206.83)** |
| **2013** | **0.58 (0.53 to 0.64)** | **8.99 (8.8 to 9.19)** | **203.84 (202.51 to 205.17)** |
| **2014** | **0.58 (0.53 to 0.64)** | **9.31 (9.11 to 9.51)** | **200.03 (198.73 to 201.33)** |
| **2015** | **0.58 (0.53 to 0.64)** | **9.73 (9.53 to 9.93)** | **201.87 (200.58 to 203.17)** |
| **2016** | **0.64 (0.58 to 0.69)** | **10.13 (9.92 to 10.33)** | **196.87 (195.6 to 198.13)** |
| **2017** | **0.64 (0.58 to 0.69)** | **10.36 (10.15 to 10.57)** | **199.61 (198.35 to 200.87)** |
| **2018** | **0.69 (0.63 to 0.75)** | **10.84 (10.63 to 11.05)** | **199.65 (198.41 to 200.89)** |
| **2019** | **0.69 (0.63 to 0.75)** | **11.11 (10.9 to 11.33)** | **200.43 (199.2 to 201.65)** |
| **2020** | **0.8 (0.74 to 0.86)** | **13.06 (12.83 to 13.3)** | **214.68 (213.42 to 215.94)** |
| **2021** | **0.95 (0.89 to 1.02)** | **13.53 (13.3 to 13.77)** | **226.39 (225.07 to 227.72)** |
| **2022** | **0.9 (0.84 to 0.97)** | **12.86 (12.63 to 13.1)** | **211.99 (210.75 to 213.23)** |
| **2023** | **0.85 (0.79 to 0.92)** | **12.63 (12.39 to 12.86)** | **207.49 (206.27 to 208.72)** |
| **2024** | **0.8 (0.74 to 0.86)** | **12.43 (12.2 to 12.66)** | **200.09 (198.92 to 201.28)** |
| **Overall** | **0.63 (0.57 to 0.68)** | **10.97 (10.75 to 11.20)** | **240.91 (239.44 to 242.38)** |

**SUPPLEMENTARY TABLE 5:** Age-adjusted mortality rates of Coronary Artery Disease (CAD) and Heart Failure (HF) in adults in the US, 1999–2024, by Age groups

| **Age-Adjusted Rate (95% CI)** | | | | | |
| --- | --- | --- | --- | --- | --- |
| **Year** | **American** | **Asian or Pacific Islander** | **Black or African American** | **White** | **Hispanic or Latino** |
| **1999** | **52.39 (46.79 to 57.98)** | **37.47 (35.35 to 39.6)** | **60.25 (58.97 to 61.53)** | **73.24 (72.81 to 73.66)** | **54.17 (52.44 to 55.9)** |
| **2000** | **47.66 (42.61 to 52.72)** | **33.39 (31.45 to 35.33)** | **60.81 (59.53 to 62.09)** | **71.62 (71.2 to 72.03)** | **52.11 (50.46 to 53.76)** |
| **2001** | **41.41 (36.75 to 46.06)** | **32.68 (30.85 to 34.5)** | **56.73 (55.51 to 57.96)** | **68.98 (68.58 to 69.39)** | **51.19 (49.6 to 52.78)** |
| **2002** | **49.73 (44.71 to 54.74)** | **31.17 (29.45 to 32.89)** | **56.34 (55.12 to 57.55)** | **66.54 (66.14 to 66.93)** | **48.26 (46.76 to 49.77)** |
| **2003** | **54.06 (48.88 to 59.24)** | **30.81 (29.15 to 32.47)** | **54.2 (53.01 to 55.38)** | **64.63 (64.25 to 65.02)** | **47.14 (45.7 to 48.59)** |
| **2004** | **51.44 (46.49 to 56.4)** | **29.93 (28.35 to 31.52)** | **51.56 (50.42 to 52.71)** | **61.47 (61.1 to 61.85)** | **43.93 (42.56 to 45.29)** |
| **2005** | **40.88 (36.63 to 45.13)** | **29.97 (28.45 to 31.49)** | **52.8 (51.66 to 53.95)** | **61.18 (60.81 to 61.55)** | **44.24 (42.91 to 45.56)** |
| **2006** | **45.77 (41.35 to 50.18)** | **28.94 (27.49 to 30.38)** | **48.65 (47.57 to 49.74)** | **56.76 (56.4 to 57.11)** | **42.32 (41.06 to 43.58)** |
| **2007** | **39.98 (35.93 to 44.03)** | **27.33 (25.96 to 28.69)** | **46.08 (45.04 to 47.12)** | **53.3 (52.97 to 53.64)** | **39.38 (38.19 to 40.56)** |
| **2008** | **39.74 (35.79 to 43.7)** | **25.14 (23.87 to 26.4)** | **44.05 (43.05 to 45.06)** | **52.26 (51.93 to 52.59)** | **37.68 (36.55 to 38.8)** |
| **2009** | **38.73 (34.95 to 42.51)** | **24.61 (23.39 to 25.82)** | **41.56 (40.6 to 42.53)** | **48.66 (48.34 to 48.98)** | **34.35 (33.31 to 35.39)** |
| **2010** | **38.87 (35.16 to 42.57)** | **23.29 (22.14 to 24.45)** | **40.81 (39.87 to 41.75)** | **47.74 (47.43 to 48.05)** | **34.17 (33.15 to 35.18)** |
| **2011** | **36.83 (33.38 to 40.27)** | **22.09 (21.02 to 23.16)** | **38.92 (38.02 to 39.82)** | **46.31 (46 to 46.61)** | **32.52 (31.57 to 33.46)** |
| **2012** | **36.13 (32.8 to 39.47)** | **21.61 (20.59 to 22.63)** | **38.24 (37.36 to 39.12)** | **44.72 (44.43 to 45.02)** | **31.23 (30.33 to 32.13)** |
| **2013** | **35.35 (32.16 to 38.55)** | **21.88 (20.9 to 22.87)** | **38.18 (37.32 to 39.03)** | **44.43 (44.13 to 44.72)** | **31.76 (30.88 to 32.64)** |
| **2014** | **37.21 (34.05 to 40.37)** | **19.98 (19.07 to 20.88)** | **38.09 (37.25 to 38.93)** | **43.81 (43.52 to 44.1)** | **31.01 (30.16 to 31.85)** |
| **2015** | **37.9 (34.8 to 41)** | **21.27 (20.37 to 22.17)** | **39.34 (38.5 to 40.18)** | **44.26 (43.97 to 44.54)** | **31.37 (30.55 to 32.19)** |
| **2016** | **37.36 (34.39 to 40.33)** | **19.67 (18.82 to 20.51)** | **38.17 (37.36 to 38.99)** | **43.57 (43.28 to 43.85)** | **30.9 (30.1 to 31.69)** |
| **2017** | **33.85 (31.11 to 36.59)** | **21.02 (20.18 to 21.86)** | **39.19 (38.38 to 40)** | **44.19 (43.91 to 44.47)** | **30.9 (30.13 to 31.67)** |
| **2018** | **36.26 (33.54 to 38.98)** | **21.28 (20.46 to 22.1)** | **38.7 (37.91 to 39.49)** | **44.48 (44.2 to 44.76)** | **31.03 (30.28 to 31.78)** |
| **2019** | **33.73 (31.15 to 36.3)** | **20.41 (19.63 to 21.2)** | **39.99 (39.2 to 40.78)** | **44.72 (44.44 to 45)** | **31.07 (30.33 to 31.81)** |
| **2020** | **39.73 (37.02 to 42.45)** | **22.85 (22.04 to 23.65)** | **46.12 (45.29 to 46.95)** | **48.07 (47.79 to 48.36)** | **35.3 (34.53 to 36.06)** |
| **2021** | **38.15 (35.44 to 41.03)** | **22.12 (21.31 to 22.96)** | **46.2 (45.35 to 47.06)** | **51.35 (51.04 to 51.65)** | **35.84 (35.07 to 36.62)** |
| **2022** | **32.96 (30.56 to 35.52)** | **21.3 (20.53 to 22.08)** | **43.75 (42.94 to 44.57)** | **48.18 (47.89 to 48.46)** | **32.13 (31.42 to 32.85)** |
| **2023** | **32.78 (30.43 to 35.27)** | **19.61 (18.89 to 20.35)** | **42.89 (42.1 to 43.69)** | **47.27 (46.98 to 47.55)** | **30.98 (30.29 to 31.68)** |
| **2024** | **32.37 (30.14 to 34.74)** | **19.18 (18.49 to 19.88)** | **41.7 (40.94 to 42.47)** | **45.71 (45.43 to 45.98)** | **28.93 (28.29 to 29.58)** |
| **Overall** | **40.04 (36.42 to 19.03)** | **24.96 (23.77 to 26.14)** | **45.51 (44.54 to 46.47)** | **52.59 (52.26 to 52.91)** | **37.45 (36.4 to 38.5)** |

**SUPPLEMENTARY TABLE 6:** Age-adjusted mortality rates of Coronary Artery Disease (CAD) and Heart Failure (HF) in adults in the US, 1999–2024, by Race

| **Age-Adjusted Rate (95% CI)** | | | | |
| --- | --- | --- | --- | --- |
| **Year** | **Northeast** | **Midwest** | **South** | **West** |
| **1999** | **69.52 (68.68 to 70.36)** | **76.37 (75.54 to 77.2)** | **69.77 (69.11 to 70.43)** | **70.96 (70.06 to 71.85)** |
| **2000** | **68.23 (67.4 to 69.06)** | **74.18 (73.37 to 74.99)** | **69.35 (68.69 to 70.01)** | **67.88 (67.02 to 68.75)** |
| **2001** | **65.62 (64.81 to 66.42)** | **70.41 (69.63 to 71.2)** | **66.72 (66.08 to 67.36)** | **65.8 (64.96 to 66.65)** |
| **2002** | **62.41 (61.63 to 63.19)** | **68.45 (67.67 to 69.22)** | **64.3 (63.68 to 64.93)** | **64.51 (63.69 to 65.33)** |
| **2003** | **60.66 (59.9 to 61.43)** | **65.56 (64.81 to 66.31)** | **62.56 (61.95 to 63.17)** | **63.42 (62.61 to 64.22)** |
| **2004** | **58.35 (57.61 to 59.1)** | **62.33 (61.6 to 63.06)** | **59.34 (58.75 to 59.93)** | **59.88 (59.1 to 60.65)** |
| **2005** | **57.7 (56.96 to 58.43)** | **62.71 (61.98 to 63.43)** | **59.45 (58.87 to 60.04)** | **58.52 (57.77 to 59.28)** |
| **2006** | **52.56 (51.86 to 53.26)** | **58.58 (57.88 to 59.27)** | **54.89 (54.33 to 55.44)** | **55.41 (54.69 to 56.14)** |
| **2007** | **49.42 (48.75 to 50.1)** | **54.86 (54.19 to 55.53)** | **51.58 (51.05 to 52.11)** | **52.11 (51.42 to 52.8)** |
| **2008** | **47.82 (47.16 to 48.47)** | **54.36 (53.7 to 55.02)** | **49.58 (49.06 to 50.09)** | **51.78 (51.1 to 52.46)** |
| **2009** | **44.13 (43.51 to 44.76)** | **50.15 (49.52 to 50.78)** | **47.11 (46.61 to 47.61)** | **47.82 (47.18 to 48.47)** |
| **2010** | **44.06 (43.44 to 44.69)** | **49.1 (48.48 to 49.72)** | **46.06 (45.58 to 46.55)** | **46.46 (45.83 to 47.09)** |
| **2011** | **43.24 (42.62 to 43.85)** | **48.32 (47.71 to 48.93)** | **43.28 (42.82 to 43.74)** | **45.46 (44.85 to 46.08)** |
| **2012** | **41.5 (40.9 to 42.09)** | **46.17 (45.58 to 46.76)** | **42.95 (42.49 to 43.4)** | **42.89 (42.3 to 43.47)** |
| **2013** | **40.96 (40.37 to 41.55)** | **45.76 (45.17 to 46.34)** | **42.86 (42.41 to 43.31)** | **42.57 (41.99 to 43.15)** |
| **2014** | **40.15 (39.57 to 40.73)** | **45.1 (44.52 to 45.68)** | **42.59 (42.15 to 43.03)** | **41.61 (41.05 to 42.17)** |
| **2015** | **39.85 (39.28 to 40.43)** | **45.73 (45.16 to 46.31)** | **43.24 (42.8 to 43.68)** | **42.39 (41.83 to 42.95)** |
| **2016** | **38.11 (37.55 to 38.67)** | **44.33 (43.77 to 44.9)** | **42.61 (42.18 to 43.04)** | **42.61 (42.06 to 43.16)** |
| **2017** | **37.63 (37.08 to 38.18)** | **46.59 (46.02 to 47.16)** | **43.3 (42.87 to 43.73)** | **42.46 (41.92 to 43.01)** |
| **2018** | **38.2 (37.65 to 38.75)** | **46.8 (46.24 to 47.37)** | **43.81 (43.39 to 44.23)** | **41.8 (41.27 to 42.33)** |
| **2019** | **37.57 (37.03 to 38.1)** | **46.63 (46.06 to 47.19)** | **44.85 (44.43 to 45.28)** | **41.91 (41.38 to 42.44)** |
| **2020** | **40.39 (39.83 to 40.94)** | **51.97 (51.38 to 52.56)** | **48.12 (47.69 to 48.55)** | **44.71 (44.17 to 45.25)** |
| **2021** | **39.77 (39.21 to 40.34)** | **53.99 (53.37 to 54.62)** | **52.11 (51.65 to 52.57)** | **48 (47.43 to 48.58)** |
| **2022** | **37.19 (36.66 to 37.72)** | **50.97 (50.38 to 51.56)** | **49.06 (48.63 to 49.49)** | **44.45 (43.92 to 44.99)** |
| **2023** | **36.28 (35.75 to 36.81)** | **50.42 (49.84 to 51.01)** | **48.18 (47.76 to 48.61)** | **42.83 (42.31 to 43.36)** |
| **2024** | **34.29 (33.8 to 34.8)** | **49.59 (49.02 to 50.17)** | **46.77 (46.36 to 47.18)** | **40.84 (40.34 to 41.35)** |
| **Overall** | **47.13 (46.50 to 47.77)** | **54.59 (53.94 to 55.24)** | **51.32 (50.82 to 51.82)** | **50.34 (49.7 to 50.99)** |

**SUPPLEMENTARY TABLE 7:** Age-adjusted mortality rates of Coronary Artery Disease (CAD) and Heart Failure (HF) in adults in the US, 1999–2024, by Census region

| **Age-Adjusted Rate (95% CI)** | | |
| --- | --- | --- |
| **Year** | **Rural** | **Urban** |
| **1999** | **83.12 (82.15 to 84.09)** | **68.87 (68.44 to 69.3)** |
| **2000** | **81.79 (80.84 to 82.75)** | **67.24 (66.82 to 67.67)** |
| **2001** | **78.03 (77.1 to 78.96)** | **64.76 (64.35 to 65.17)** |
| **2002** | **75.6 (74.68 to 76.51)** | **62.55 (62.15 to 62.95)** |
| **2003** | **75.19 (74.28 to 76.1)** | **60.39 (60 to 60.78)** |
| **2004** | **72.05 (71.17 to 72.94)** | **57.34 (56.96 to 57.72)** |
| **2005** | **72.41 (71.53 to 73.29)** | **56.93 (56.56 to 57.31)** |
| **2006** | **66.92 (66.08 to 67.76)** | **52.96 (52.6 to 53.32)** |
| **2007** | **63.33 (62.52 to 64.15)** | **49.63 (49.29 to 49.97)** |
| **2008** | **63.17 (62.36 to 63.97)** | **48.23 (47.9 to 48.56)** |
| **2009** | **59.36 (58.58 to 60.14)** | **44.87 (44.56 to 45.19)** |
| **2010** | **57.66 (56.9 to 58.42)** | **44.13 (43.82 to 44.45)** |
| **2011** | **55.87 (55.13 to 56.62)** | **42.71 (42.41 to 43.02)** |
| **2012** | **54.1 (53.37 to 54.82)** | **41.27 (40.97 to 41.56)** |
| **2013** | **53.18 (52.46 to 53.9)** | **41.1 (40.81 to 41.39)** |
| **2014** | **53.17 (52.46 to 53.88)** | **40.36 (40.08 to 40.64)** |
| **2015** | **54.65 (53.94 to 55.37)** | **40.69 (40.41 to 40.97)** |
| **2016** | **53.54 (52.83 to 54.24)** | **39.93 (39.65 to 40.2)** |
| **2017** | **54.48 (53.77 to 55.19)** | **40.53 (40.25 to 40.8)** |
| **2018** | **55.13 (54.42 to 55.84)** | **40.65 (40.37 to 40.92)** |
| **2019** | **56.18 (55.47 to 56.89)** | **40.73 (40.46 to 41)** |
| **2020** | **60.92 (60.19 to 61.66)** | **44.07 (43.79 to 44.34)** |
| **Overall** | **63.62 (62.82 to 64.43)** | **49.54 (49.21 to 49.87)** |

**SUPPLEMENTARY TABLE 8:** Age-adjusted mortality rates of Coronary Artery Disease (CAD) and Heart Failure (HF) in adults in the US, 1999–2020, by Urbanization

| **Age-Adjusted Rate (95% CI)** | | |
| --- | --- | --- |
| **State** | **1999 - 2020** | **2021-2024** |
| **Alabama** | **44.81 (44.32 to 45.29)** | **33.86 (32.98 to 34.77)** |
| **Alaska** | **36.13 (34.49 to 37.78)** | **34.78 (31.87 to 37.9)** |
| **Arizona** | **43.87 (43.45 to 44.28)** | **39.25 (38.49 to 40.03)** |
| **Arkansas** | **69.98 (63.25 to 64.70)** | **66.14 (64.54 to 67.77)** |
| **California** | **50.88 (50.69 to 51.08)** | **37.81 (37.46 to 38.16)** |
| **Colorado** | **48.78 (48.23 to 49.32)** | **48.34 (47.26 to 49.44)** |
| **Connecticut** | **40.64 (40.15 to 41.13)** | **23.44 (22.62 to 24.3)** |
| **Delaware** | **51.16 (49.99 to 52.34)** | **53.62 (51.26 to 56.08)** |
| **District of Columbia** | **36.98 (35.68 to 38.27)** | **35.27 (32.52 to 38.2)** |
| **Florida** | **41.15 (40.95 to 41.35)** | **43.32 (42.9 to 43.75)** |
| **Georgia** | **39.77 (39.40 to 40.13)** | **34.33 (33.66 to 35.01)** |
| **Hawaii** | **30.38 (29.68 to 31.09)** | **32.37 (30.91 to 33.9)** |
| **Idaho** | **53.78 (52.81 to 54.75)** | **57.84 (55.86 to 59.87)** |
| **Illinois** | **44.74 (44.44 to 45.04)** | **34.93 (34.36 to 35.51)** |
| **Indiana** | **59.56 (59.08 to 60.05)** | **56.72 (55.71 to 57.74)** |
| **Iowa** | **62.83 (62.18 to 63.47)** | **59.18 (57.76 to 60.63)** |
| **Kansas** | **51.57 (50.92 to 52.22)** | **52.9 (51.45 to 54.39)** |
| **Kentucky** | **59.92 (58.53 to 59.72)** | **58.54 (57.28 to 59.82)** |
| **Louisiana** | **45.08 (44.50 to 45.60)** | **46.99 (45.86 to 48.14)** |
| **Maine** | **53.54 (52.63 to 54.45)** | **52.83 (50.92 to 54.8)** |
| **Maryland** | **40.38 (39.95 to 40.82)** | **43.47 (42.55 to 44.4)** |
| **Massachusetts** | **39.10 (38.74 to 39.46)** | **34.68 (33.94 to 35.44)** |
| **Michigan** | **54.24 (53.88 to 54.60)** | **52.9 (52.12 to 53.69)** |
| **Minnesota** | **45.73 (45.28 to 46.19)** | **60.77 (59.66 to 61.9)** |
| **Mississippi** | **66.62 (65.85 to 67.39)** | **65.12 (63.47 to 66.8)** |
| **Missouri** | **51.25 (50.80 to 51.70)** | **43.89 (42.99 to 44.81)** |
| **Montana** | **41.52 (40.55 to 42.49)** | **46.31 (44.18 to 48.53)** |
| **Nebraska** | **54.36 (53.54 to 55.18)** | **59.85 (57.94 to 61.81)** |
| **Nevada** | **34.87 (34.23 to 35.52)** | **46.71 (45.32 to 48.14)** |
| **New Hampshire** | **52.22 (51.24 to 53.19)** | **44.71 (42.88 to 46.61)** |
| **New Jersey** | **44.45 (44.11 to 44.80)** | **29.6 (29 to 30.21)** |
| **New Mexico** | **42.81 (42.06 to 43.55)** | **38.62 (37.2 to 40.1)** |
| **New York** | **48.51 (48.27 to 48.75)** | **34.24 (33.81 to 34.68)** |
| **North Carolina** | **47.87 (47.50 to 48.23)** | **48.18 (47.44 to 48.94)** |
| **North Dakota** | **64.11(62.71 to 65.50)** | **51.35 (48.59 to 54.24)** |
| **Ohio** | **66.09 (65.72 to 66.45)** | **52.42 (51.71 to 53.14)** |
| **Oklahoma** | **76.84 (76.12 to 77.56)** | **73.05 (71.54 to 74.6)** |
| **Oregon** | **58.15 (57.55 to 58.74)** | **66.92 (65.58 to 68.28)** |
| **Pennsylvania** | **52.57 (52.28 to 52.86)** | **44.47 (43.88 to 45.07)** |
| **Rhode Island** | **71.59 (70.41 to 72.77)** | **56.83 (54.51 to 59.25)** |
| **South Carolina** | **54.41 (50.87 to 51.95)** | **55.61 (54.5 to 56.74)** |
| **South Dakota** | **66.95 (65.64 to 68.27)** | **70.06 (67.08 to 73.16)** |
| **Tennessee** | **69.55 (69.01 to 70.08)** | **63.08 (62.02 to 64.15)** |
| **Texas** | **57.21 (56.94 to 57.49)** | **51.59 (51.07 to 52.11)** |
| **Utah** | **41.77 (41.01 to 42.52)** | **45.7 (44.12 to 47.33)** |
| **Vermont** | **64.38 (62.85 to 65.90)** | **61.28 (58.19 to 64.54)** |
| **Virginia** | **38.67 (38.31 to 39.04)** | **44.78 (43.98 to 45.59)** |
| **Washington** | **63.14 (62.64 to 63.64)** | **63.44 (62.42 to 64.48)** |
| **West Virginia** | **76.92 (75.98 to 77.87)** | **63.24 (61.34 to 65.19)** |
| **Wisconsin** | **48.50 (48.06 to 48.94)** | **60.8 (59.72 to 61.9)** |
| **Wyoming** | **47.83 (46.31 to 49.36)** | **53.35 (50.06 to 56.82)** |

**SUPPLEMENTARY TABLE 9:** Age-adjusted mortality rates of Coronary Artery Disease (CAD) and Heart Failure (HF) in adults in the US, 1999–2020 and 2021-2024, by States.

| **Age-Adjusted Rate (95% CI)** | | |
| --- | --- | --- |
| **Year** | **Chronic Ischemic Cardiomyopathy** | **Acute MI** |
| **1999** | **5.45 (5.34 to 5.56)** | **15.33 (15.15 to 15.52)** |
| **2000** | **5.24 (5.13 to 5.34)** | **15.12 (14.94 to 15.3)** |
| **2001** | **5.09 (4.99 to 5.19)** | **14.27 (14.09 to 14.44)** |
| **2002** | **5.03 (4.93 to 5.13)** | **13.75 (13.58 to 13.92)** |
| **2003** | **5.01 (4.91 to 5.11)** | **13.09 (12.93 to 13.26)** |
| **2004** | **4.9 (4.8 to 5)** | **12.26 (12.11 to 12.42)** |
| **2005** | **4.99 (4.89 to 5.09)** | **11.85 (11.69 to 12)** |
| **2006** | **4.49 (4.39 to 4.58)** | **10.86 (10.71 to 11.01)** |
| **2007** | **4.28 (4.19 to 4.37)** | **10.07 (9.93 to 10.21)** |
| **2008** | **4.37 (4.28 to 4.46)** | **9.69 (9.55 to 9.82)** |
| **2009** | **4.25 (4.16 to 4.34)** | **8.92 (8.8 to 9.05)** |
| **2010** | **4.23 (4.14 to 4.31)** | **8.55 (8.43 to 8.68)** |
| **2011** | **4.04 (3.95 to 4.12)** | **8.25 (8.13 to 8.37)** |
| **2012** | **3.94 (3.86 to 4.02)** | **8 (7.89 to 8.12)** |
| **2013** | **3.83 (3.75 to 3.91)** | **7.79 (7.67 to 7.9)** |
| **2014** | **3.87 (3.79 to 3.95)** | **7.73 (7.62 to 7.85)** |
| **2015** | **4.02 (3.94 to 4.11)** | **7.76 (7.65 to 7.87)** |
| **2016** | **4.19 (4.11 to 4.27)** | **7.73 (7.62 to 7.84)** |
| **2017** | **4.32 (4.23 to 4.4)** | **7.85 (7.74 to 7.96)** |
| **2018** | **4.16 (4.08 to 4.24)** | **7.82 (7.71 to 7.93)** |
| **2019** | **4.07 (4 to 4.15)** | **7.76 (7.65 to 7.86)** |
| **2020** | **4.32 (4.24 to 4.4)** | **8.35 (8.24 to 8.46)** |
| **2021** | **4.46 (4.38 to 4.55)** | **9.18 (9.06 to 9.3)** |
| **2022** | **4.27 (4.19 to 4.34)** | **8.54 (8.43 to 8.65)** |
| **2023** | **4.2 (4.12 to 4.28)** | **8.06 (7.96 to 8.17)** |
| **2024** | **3.87 (3.8 to 3.94)** | **7.76 (7.65 to 7.86)** |
| **Overall** | **4.41 (4.33 to 4.50)** | **9.85 (9.72 to 9.99** |

**SUPPLEMENTARY TABLE 10:** Age-adjusted mortality rates of Coronary Artery Disease (CAD) and Heart Failure (HF) in adults in the US, 1999–2024, by Clinical Presentation

| **Age Adjusted Rate (95% CI)** | | |
| --- | --- | --- |
| **Year** | **CAD-HF ( Without COVID Deaths)** | **CAD-HF (with COVID Deaths)** |
| **2018** | **43.06 (42.81 to 43.32)** | **43.06 (42.81 to 43.32)** |
| **2019** | **43.31 (43.06 to 43.56)** | **43.31 (43.05 to 43.56)** |
| **2020** | **44.68 (44.43 to 44.94)** | **46.81 (46.55 to 47.07)** |
| **2021** | **47.19 (46.92 to 47.45)** | **49.33 (49.06 to 49.61)** |
| **2022** | **44.94 (44.69 to 45.2)** | **46.26 (46.01 to 46.52)** |
| **2023** | **44.77 (44.52 to 45.03)** | **45.28 (45.03 to 45.54)** |
| **2024** | **43.43 (43.18 to 43.67)** | **43.74 (43.5 to 43.99)** |

**SUPPLEMENTARY TABLE 11:** Age-adjusted mortality rates of Coronary Artery Disease (CAD) and Heart Failure (HF) in adults in the US, 2018-2024, by sensitivity analysis With Covid deaths VS without Covid deaths.

| **Age Adjusted Rate (95% CI)** | | |
| --- | --- | --- |
| **Year** | **Without Hypertensive HF** | **With Hypertensive HF** |
| **1999** | 71.61 (71.22 to 72.01) | 71.62 (71.22 to 72.01) |
| **2000** | 69.31 (68.92 to 69.69) | 70.03 (69.64 to 70.41) |
| **2001** | 66.6 (66.23 to 66.98) | 67.28 (66.9 to 67.66) |
| **2002** | 64.43 (64.06 to 64.8) | 65.03 (64.66 to 65.4) |
| **2003** | 62.54 (62.18 to 62.9) | 63.14 (62.78 to 63.5) |
| **2004** | 59.48 (59.13 to 59.82) | 60.08 (59.73 to 60.43) |
| **2005** | 59.22 (58.87 to 59.56) | 59.78 (59.44 to 60.13) |
| **2006** | 54.92 (54.59 to 55.25) | 55.52 (55.19 to 55.85) |
| **2007** | 51.59 (51.28 to 51.91) | 52.13 (51.82 to 52.45) |
| **2008** | 50.37 (50.06 to 50.67) | 50.91 (50.6 to 51.22) |
| **2009** | 46.91 (46.61 to 47.2) | 47.49 (47.19 to 47.78) |
| **2010** | 45.99 (45.7 to 46.28) | 46.53 (46.24 to 46.82) |
| **2011** | 44.45 (44.17 to 44.73) | 45.03 (44.75 to 45.32) |
| **2012** | 42.95 (42.68 to 43.22) | 43.49 (43.22 to 43.76) |
| **2013** | 42.65 (42.38 to 42.91) | 43.2 (42.93 to 43.47) |
| **2014** | 41.92 (41.66 to 42.18) | 42.56 (42.3 to 42.83) |
| **2015** | 42.39 (42.13 to 42.66) | 43.06 (42.8 to 43.33) |
| **2016** | 41.47 (41.21 to 41.72) | 42.25 (41.99 to 42.51) |
| **2017** | 41.94 (41.68 to 42.19) | 42.86 (42.61 to 43.12) |
| **2018** | 41.99 (41.74 to 42.24) | 43.06 (42.81 to 43.32) |
| **2019** | 41.93 (41.68 to 42.17) | 43.31 (43.05 to 43.56) |
| **2020** | 44.95 (44.7 to 45.21) | 46.81 (46.55 to 47.07) |
| **2021** | 47.27 (47.01 to 47.54) | 49.33 (49.06 to 49.61) |
| **2022** | 44.3 (44.05 to 44.55) | 46.26 (46.01 to 46.52) |
| **2023** | 43.25 (43 to 43.5) | 45.28 (45.03 to 45.54) |
| **2024** | 41.57 (41.33 to 41.81) | 43.74 (43.5 to 43.99) |
| **Overall** | 50.23 (49.93 to 50.52) | 51.14 (50.85 to 51.45) |

**SUPPLEMENTARY TABLE 12:** Age-adjusted mortality rates of Coronary Artery Disease (CAD) and Heart Failure (HF) in adults in the US, 1999–2024, by sensitivity analysis without hypertensive HF VS with Hypertensive HF.
